# SAPS3 subunit of protein phosphatase 6 is an AMPK inhibitor and controls metabolic homeostasis upon dietary challenge in male mice

**DOI:** 10.1038/s41467-023-36809-1

**Published:** 2023-03-13

**Authors:** Ying Yang, Michael A. Reid, Eric A. Hanse, Haiqing Li, Yuanding Li, Bryan I. Ruiz, Qi Fan, Mei Kong

**Affiliations:** 1 Department of Molecular Biology and Biochemistry; School of Biological Sciences, University of California, Irvine, Irvine, CA 92697 USA; 2 Department of Cancer Biology, Beckman Research Institute of City of Hope National Medical Center, Duarte, CA 91010 USA; 3 Integrative Genomics Core, Beckman Research Institute, City of Hope National Medical Center, Duarte, CA 91010 USA

**Keywords:** Cell signalling, Metabolism, Metabolic syndrome

## Abstract

Inhibition of AMPK is tightly associated with metabolic perturbations upon over nutrition, yet the molecular mechanisms underlying are not clear. Here, we demonstrate the serine/threonine-protein phosphatase 6 regulatory subunit 3, SAPS3, is a negative regulator of AMPK. SAPS3 is induced under high fat diet (HFD) and recruits the PP6 catalytic subunit to deactivate phosphorylated-AMPK, thereby inhibiting AMPK-controlled metabolic pathways. Either whole-body or liver-specific deletion of SAPS3 protects male mice against HFD-induced detrimental consequences and reverses HFD-induced metabolic and transcriptional alterations while loss of SAPS3 has no effects on mice under balanced diets. Furthermore, genetic inhibition of AMPK is sufficient to block the protective phenotype in SAPS3 knockout mice under HFD. Together, our results reveal that SAPS3 is a negative regulator of AMPK and suppression of SAPS3 functions as a guardian when metabolism is perturbed and represents a potential therapeutic strategy to treat metabolic syndromes.

## Abstract (web summary)

Inhibition of AMPK leads to metabolic perturbations yet how AMPK is inactivated is not fully understood. Here the authors show protein phosphatase 6 subunit SAPS3 is a negative regulator of AMPK and loss of SAPS3 activates AMPK and protects male mice against overnutrition.

## Introduction

Metabolic syndromes, cancer, and ageing, which are all associated with deterioration in the maintenance of metabolic homeostasis, are fundamental health problems worldwide. The AMP-activated protein kinase (AMPK) senses metabolic stress and is a central mediator in maintaining metabolic homeostasis within cells and at the whole-organism level. AMPK relays the state of the cells’ AMP: ATP and ADP: ATP ratios and its activation promotes restoration of cellular energy through the rapid reduction in macromolecular synthesis, enhancement of glucose uptake, and fatty acid oxidation^1^. Systemically, AMPK has adapted to integrate hormone signals to balance organismal energy intake and expenditure at the whole-body level^2^. Thus, AMPK activation has become an attractive target for treating diseases associated with metabolic perturbations, such as diabetes, obesity, fatty liver disease, cancer, and ageing^3–5^. However, little is known about regulators of AMPK that antagonize the AMPK activators. These factors are critical to efficiently target AMPK activation for therapies.

While it has been demonstrated that AMPK activity is tightly regulated by reversible protein phosphorylation, how AMPK is dephosphorylated and inactivated upon recovery from metabolic stress is much less clear. We and others have indicated that the protein phosphatase 2 A (PP2A) family phosphatases may contribute to AMPK dephosphorylation^6–8^. However, a specific phosphatase complex among the hundreds of possible PP2A family phosphatase complexes that directly dephosphorylates AMPK and regulates metabolism in vivo has not been identified. Unlike kinases, serine/threonine phosphatase activity is promiscuous and phosphatase specificity is governed largely by associated proteins. The PP2A family protein phosphatases (including PP2A, PP4, and PP6) play an important role in many cellular functions^9,10^. The PP2A family catalytic subunits (C) are among the most conserved enzymes in eukaryotic cells and dephosphorylate serine and threonine residues without specificity for substrates. Thus, the specificity of the phosphatase is conferred by association with different regulatory subunits^9–11^. To identify specific protein phosphatase complexes that bind to AMPK, we performed protein mass spectrometric analysis using Flag-tagged AMPKα and found components of the PP6 phosphatase complex, including the regulatory subunit SAPS3 and the catalytic subunit of PP6 in association with AMPK. SAPS3 is one of the three PP6 regulatory subunits and plays a critical role in determining PP6 substrate specificity^12,13^. However, the biological functions of SAPS3 are largely unknown. Interestingly, it has been reported in yeast the Sit4 phosphatase (the catalytic subunit of PP6 homolog) and Snf1 kinase (AMPK homolog) have opposing functions in regulating the transcriptional response during nutrient-sensing^14^, suggesting PP6 complex could be an evolutionally conserved negative regulator of AMPK.

Here, we show SAPS3 is upregulated upon over nutrition and facilitates PP6C binding to and dephosphorylates AMPK. To evaluate the role of SAPS3 in vivo, we generated SAPS3 whole-body knockout mice and observed loss of SAPS3 leads to AMPK activation in vivo and maintains glucose homeostasis under high fat diet. Moreover, specific deletion of SAPS3 in the liver is sufficient to reverse the adverse effects precipitated by HFD. Following metabolomic and gene expression profiling, we identify global metabolic and transcriptional responses under HFD are reversed in liver-specific SAPS3 knockout mice. Furthermore, blocking AMPK diminishes the protective effects found in SAPS3 knockout mice under HFD. In summary, we demonstrate targeting SAPS3 might be an efficient strategy to restore metabolic homeostasis and treat diseases associated with metabolic perturbations.

## Results

### SAPS3 brings the PP6 catalytic subunit to dephosphorylate AMPK

**Fig. 1:**
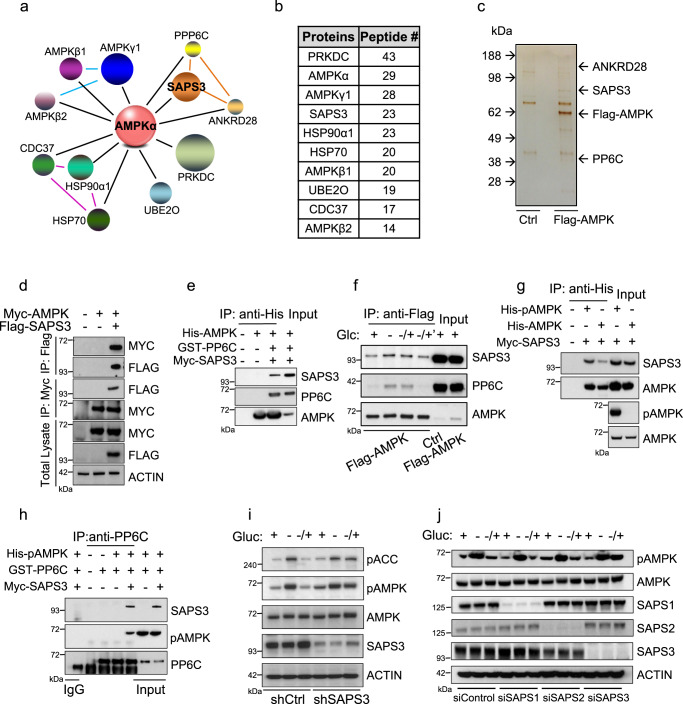
SAPS3 brings the PP6 catalytic subunit to dephosphorylate AMPK. **a** Schematic model of top binding proteins to AMPK by mass spectrometry. The bigger sphere represents more binding peptides to AMPK. Colored lines represent previously reported interactions. PRKDC, DNA-dependent protein kinase catalytic subunit; HSP90α1, heat shock protein 90 alpha 1; HSP70, heat shock protein 70; UBE2O, ubiquitin-conjugating enzyme E2 O; CDC37, cell division cycle 37; ANKRD28, ankyrin repeat domain 28. **b** The top binding proteins to AMPK by mass spectrometry. **c** 293 T cells were transfected with Flag-AMPK and treated with glucose deprivation and addback. Cell lysates were purified with anti-Flag beads and followed by silver staining. The result is representative of three independent experiments. **d** Flag-SAPS3 and Myc-AMPK were co-expressed in 293 T cells. Cell lysates were immunoprecipitated with anti-Myc or anti-Flag beads followed by Western blotting. The results are representative of three independent experiments. **e** His-tag pull down of AMPK was performed followed by Western blotting using antibodies indicated. The results are representative of three independent experiments. **f** Flag-AMPK was overexpressed in 293 T cells that were then treated with glucose deprivation (−), addback (−/+) 1 h, or addback (−/+) 3 h. Cell lysates were immunoprecipitated using anti-flag beads followed by Western blotting. The results are representative of three independent experiments. **g** His-tag pull down of pAMPK or AMPK was performed, followed by Western blotting. The results are representative of three independent experiments. **h** Purified protein PP6C was used to pull down SAPS3 and pAMPK. The results are representative of three independent experiments. **i** HT1080 cells expressing either control (Ctrl) or SAPS3 shRNA were cultured in glucose-free medium and addback (−/+) and cell lysates were analyzed by Western blotting. The results are representative of three independent experiments. **j** HT1080 cells were transfected with siControl, siSAPS1, siSAPS2, or siSAPS3 respectively. Cells were cultured in the glucose-free medium, and glucose was added back (−/+) and cell lysates were analyzed by Western blotting. The results are representative of three independent experiments.

AMPK is rapidly phosphorylated upon glucose deprivation and resupplying glucose causes massive dephosphorylation of AMPK, suggesting the activity of nutrient-mediated phosphatases (Supplementary Fig. 1a, b). To identify specific protein phosphatase complexes that bind to AMPK, we performed protein mass spectrometric analysis using Flag-tagged AMPKα expressed in 293 T cells following glucose deprivation and resupply. Among the identified proteins, AMPK subunits β and γ were identified, thus validating the approach (Fig. 1a). Besides most identified candidates being reported AMPK binding proteins^15–17^, we observed the most abundant peptides, other than AMPK subunits, were from SAPS3 (Fig. 1b). The catalytic subunit PP6C and the scaffolding subunit of PP6, ANKRD28, were also identified in the complex, suggesting the SAPS3-containing PP6 holoenzyme may play a role in AMPK dephosphorylation. We further validated the interaction of SAPS3 and AMPK in 293 T cells using co-immunoprecipitation/co-transfection (Fig. 1c, d). Moreover, we found that endogenous SAPS3 colocalized with AMPK in HT1080 cells (Supplementary Fig. 1c). Using recombinant proteins, we found that SAPS3 can directly bind to AMPK in vitro (Fig. 1e).

We next sought to determine if the SAPS3-containing PP6 phosphatase complex dephosphorylates AMPK. First, we examined whether the association of SAPS3/PP6C with AMPK is responsive to glucose. Interestingly, binding of the SAPS3/PP6 complex with AMPK started to accumulate after glucose withdrawal and reached maximal level upon short-term glucose addback. The complex dissociated after the return of glucose for 3 h indicating the phosphatase complex is assembled when AMPK is phosphorylated, perhaps in preparation for nutrient recovery and subsequent dephosphorylation (Fig. 1f). We next tested whether SAPS3 had a preference for the phosphorylated form of AMPK and found that SAPS3 preferably associated with phospho-AMPK (Fig. 1g). To gain more insight into the dynamics of this complex, we used recombinant protein binding assays and found that SAPS3 was required for the association of PP6C and AMPK (Fig. 1h). These data suggest that SAPS3 is essential for bringing the PP6 catalytic subunit to AMPK.

Next, we investigated whether SAPS3 affects AMPK signaling. We found that knockdown of SAPS3 using shRNA significantly decreased dephosphorylation of both AMPK and its downstream substrate, ACC, upon glucose addback (Fig. 1i and Supplementary Fig. 1d, e). SAPS3 is part of a family that includes three members, SAPS1, SAPS2, and SAPS3. To identify whether these other family members play a role in the mediation of AMPK, we knocked down SAPS1, SAPS2, or SAPS3 and found only knockdown of SAPS3 was able to modulate AMPK dephosphorylation upon glucose addback (Fig. 1j and Supplementary Fig. 1f).

### SAPS3 deletion in mice reverses HFD-induced detrimental effects

**Fig. 2:**
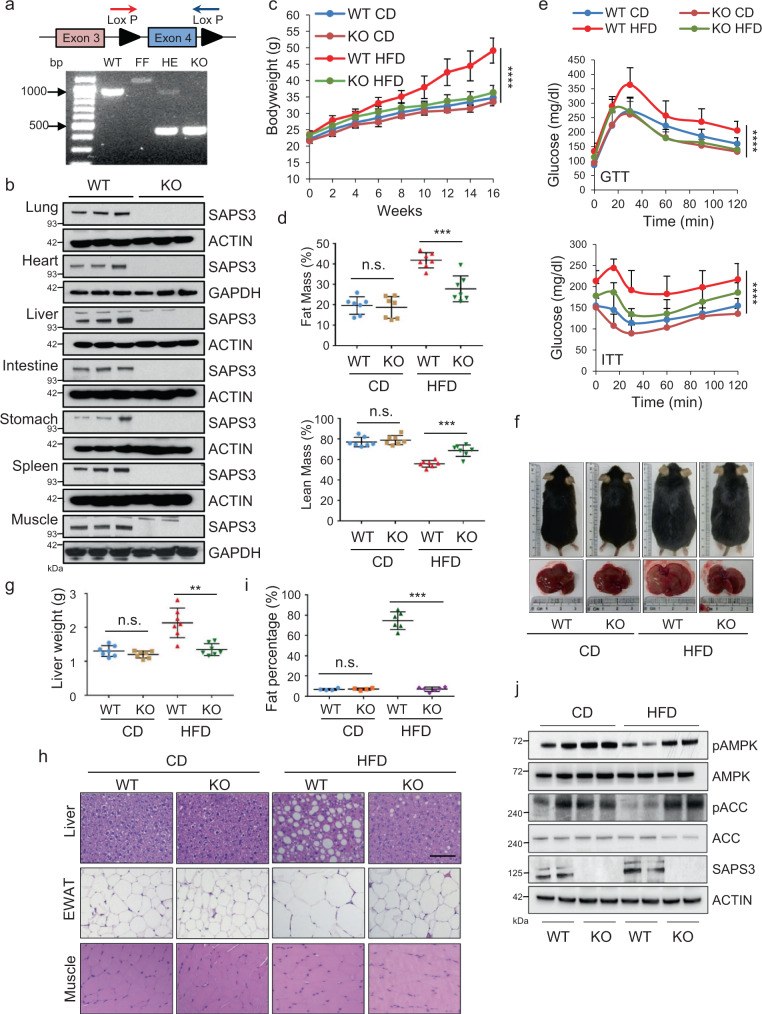
SAPS3 deletion in mice reverses HFD induced detrimental effects. **a** Schematic strategy of *ppp6r3* knockout. WT: *ppp6r3*^*+/+*^; FF: *ppp6r3* ^*fl/fl*^; HE: *ppp6r3*^*+/-*^; KO: *ppp6r3*^*-/-*^. Colored arrows indicated the positions of primers designed to determine the genotype of wildtype (WT) and SAPS3 knockout (KO) mice. PCR analysis showed a complete loss of SAPS3 expression. The results are representative of at least three independent experiments. **b** Indicated tissues were collected from SAPS3 KO mice. Tissue lysates were analyzed by Western blotting. *n* = 3 mice per group. **c** 8 weeks old male WT and KO mice were fed with HFD (45 kcal% fat) for 16 weeks. Bodyweight was measured (CD, ctrl diet; HFD, high-fat diet). Mean ± s.d., *n* = 8 mice per group analyzed by one-way ANOVA, ****p < 0.0001. **d** Fat mass and lean mass composition of mice were measured by echo-MRI. Mean ± s.d., *n* = 7 mice per group analyzed by two-tailed *t*-test. Fat mass ***p = 0.0005, lean mass ***p = 0.0004; n.s., not significant. **e** Glucose tolerance test and insulin tolerance test were performed at the endpoint. Mean ± s.d., CD, *n* = 7 mice per group; HFD, *n* = 8 mice per group analyzed by one-way ANOVA, ****p < 0.0001. **f** Representative pictures of WT and KO mice and their livers. Liver weight was measured and shown in **g**. Mean ± s.d., *n* = 7 mice per group analyzed by two-tailed *t*-test, **p = 0.002; n.s., not significant. **h** Representative images of H&E staining of the liver, epididymal white adipose tissue (eWAT), and muscle. Scale bar, 100 µm. **i** The quantification of fat percentages from the H&E staining images shown in **h**. Mean ± s.d., CD, *n* = 4 mice per group; HFD, *n* = 6 per group analyzed by two-tailed *t*-test, ***p = 3.06E-06; n.s., not significant. **j** Liver samples were collected from WT and SAPS3 KO mice under CD and HFD. Tissue lysates were analyzed by Western blotting. The results are representative of two independent experiments.

To evaluate the biological functions of SAPS3 on glucose homeostasis in vivo, we generated SAPS3 whole-body knockout mice (SAPS3 KO). Loss of SAPS3 gene expression was confirmed by PCR (Fig. 2a), and loss of protein expression was confirmed in a variety of tissues by Western blotting (Fig. 2b). Interestingly, knockout of SAPS3 had no significant effects on mice under a control balanced diet. However, when we put these mice on an HFD with 45 kcal% fat for 16 weeks, SAPS3 KO mice significantly reduced the increase in body weight to a similar level of mice fed with the control diet (CD) (Fig. 2c) without change in food intake (Supplementary Fig. 2a). HFD-fed SAPS3 KO mice had less fat mass and more lean mass compared to WT mice using MRI analysis (Fig. 2d), indicating loss of SAPS3 prevents mice from HFD-induced obesity. To assess glucose homeostasis in these mice, we performed glucose tolerance test (GTT) and insulin tolerance test (ITT). SAPS3 knockout mice maintained lower levels of blood glucose when subjected to both GTT and ITT compared to WT mice under HFD (Fig. 2e and Supplementary Fig. 2b). Interestingly, we did not observe a significant difference in blood glucose levels between SAPS3 KO and WT mice under CD during GTT and ITT, indicating the loss of SAPS3 affects the response to over-nutrition but has no discernible effect on a balanced diet. Moreover, HFD-induced liver hypertrophy was almost completely prevented in SAPS3 KO mice (Fig. 2f, g). Unlike the liver from WT mice fed with HFD, SAPS3 KO mice showed no signs of hepatic steatosis characterized by large lipid droplets. The enlarged adipocytes from epididymal white adipose tissue (eWAT) under HFD were also reversed in SAPS3 KO mice (Fig. 2h, i). We didn’t find a significant difference in muscle cells in SAPS3 KO mice compared to WT under both CD and HFD (Fig. 2h). Consistent with our in vitro data, the deletion of SAPS3 maintained AMPK activity in liver tissues under HFD (Fig. 2j and Supplementary Fig. 2c). In addition, we found HFD substantially increased SAPS3 expression, but not SAPS1 or SAPS2, along with reducing AMPK activity (Supplementary Fig. 2d, e). Taken together, these results indicate that the loss of SAPS3 substantially reverses the detrimental phenotypes that are induced by HFD.

### Liver-specific deletion of SAPS3 is sufficient to improve the systemic response to high fat diet

**Fig. 3:**
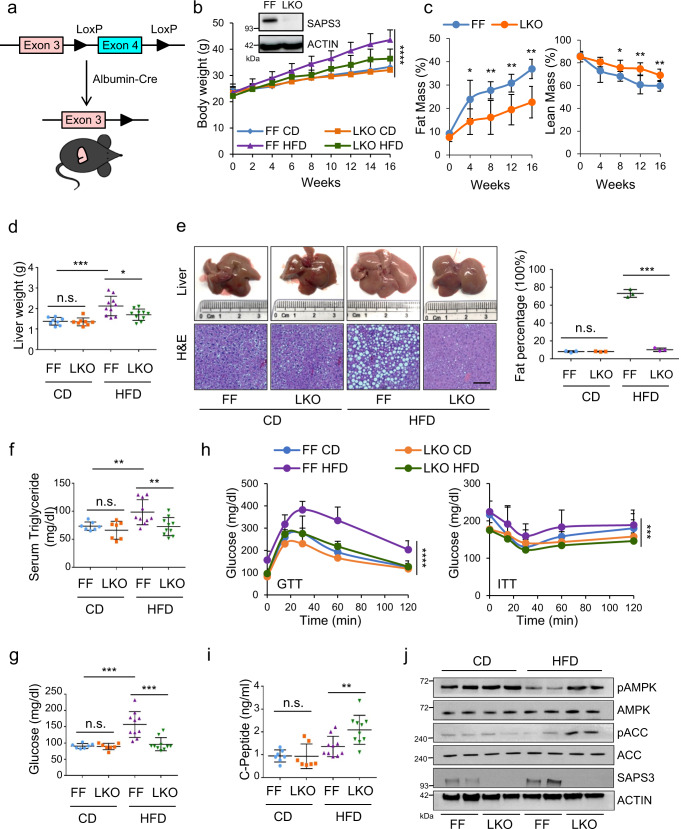
Liver specific deletion of SAPS3 is sufficient to improve the systemic response to high fat diet. **a** Schematic showing of the generation of SAPS3 liver specific knockout mice. **b** 8 weeks old male FF and LKO mice were fed with CD or HFD (32.5 kcal% fat) for 16 weeks (FF, *ppp6r3* ^*fl/fl*^ mice; LKO, SAPS3 liver specific knockout mice). Bodyweight was measured. Mean ± s.d., CD *n* = 8 mice per group; HFD *n* = 10 mice per group analyzed by one-way ANOVA, ****p < 0.0001. The SAPS3 protein expression in the liver was tested by Western blotting. The results are representative of three independent experiments. **c** Fat mass and lean mass composition of mice under HFD were measured by echo-MRI every 4 weeks. Mean ± s.d., *n* = 6 mice per group analyzed by two-tailed *t*-test. Fat mass, week: 4, *p = 0.042; 8, **p = 0.009; 12, **p = 0.005; 16, **p = 0.002. Lean mass, week: 8, *p = 0.039; 12, **p = 0.006; 16, **p = 0.009. **d** Liver weight was compared. Mean ± s.d., CD *n* = 8 mice per group; HFD *n* = 10 mice per group analyzed by two-tailed *t*-test, *p = 0.026; ***p = 0.0006; n.s., not significant. **e** Representative pictures of livers, H&E staining and quantification of liver samples after H&E staining. Scale bar, 100 µm. Mean ± s.d., *n* = 3 mice per group analyzed by two-tailed *t*-test, ***p = 0.0003; n.s., not significant. **f** Serum triglyceride level was measured. Mean ± s.d., CD *n* = 7 mice per group; HFD *n* = 10 mice per group analyzed by two-tailed *t*-test. FF (CD/HFD), **p = 0.007; HFD (FF/LKO), **p = 0.009; n.s., not significant. **g** Fasting blood glucose level was measured. Mean ± s.d., CD *n* = 7 mice per group; HFD *n* = 10 mice per group analyzed by two-tailed *t*-test. FF (CD/HFD), ***p = 0.0004; HFD (FF/LKO), ***p = 0.0008; n.s., not significant. **h** Glucose tolerance test and insulin tolerance test were performed at the endpoint. Mean ± s.d., CD *n* = 7 mice per group; HFD *n* = 10 mice per group analyzed by one-way ANOVA. GTT, ****p < 0.0001; ITT, ***p = 0.0005. **i** The concentration of C-peptide was compared between groups. Mean ± s.d., CD *n* = 7 mice per group; HFD *n* = 10 mice per group analyzed by two-tailed *t*-test, **p = 0.008; n.s., not significant. **j** Liver samples were collected from FF and LKO mice under CD and HFD. Tissue lysates were analyzed by Western blotting. The results are representative of two independent experiments.

The liver plays a key role in the regulation of whole-body energy metabolism, and targeting the liver attenuates HFD-induced obesity and diabetes^18–20^, we next crossbred *ppp6r3* ^*fl/fl*^ (FF) mice with a mouse line expressing *Cre* under the control of the liver-specific albumin promoter to generate liver-specific SAPS3 knockout mice (LKO) (Fig. 3a). To more closely model human intakes, we fed the mice using a moderately high-fat diet (32.5 kcal% fat). Deletion of SAPS3 in the liver had no noticeable effect on mice and the bodyweights of FF and LKO mice were similar over time under CD (Fig. 3b). Mirroring our results from the whole-body knock-out, we found the body weights of LKO mice were significantly lower than FF mice under HFD (Fig. 3b) with no change in food intake (Supplementary Fig. 2f), suggesting that SAPS3 deletion in the liver mitigates the effects induced by HFD. In addition, we tracked fat mass and lean mass composition during the course of the HFD and found that there was less fat and more lean mass composition in LKO mice compared to FF mice (Fig. 3c). Moreover, liver weights and tissue staining showed HFD-induced hepatic steatosis was almost completely blocked in LKO mice (Fig. 3d,e). HFD-induced increases in serum triglycerides were also significantly reduced in LKO mice (Fig. 3f).

One of the main functions of the liver is to respond to low glucose levels by triggering gluconeogenesis, a response in part mediated by AMPK^21^. Thus, we asked whether loss of SAPS3 in the liver would affect the systemic response to fasting. We found that LKO mice were not affected by fasting glucose levels under normal dietary conditions (Fig. 3g). Interestingly, LKO mice had significantly lower levels of fasting blood glucose compared to FF mice under HFD (Fig. 3g). Moreover, LKO mice under HFD cleared blood glucose more efficiently than FF mice and performed significantly better in ITT (Fig. 3h and Supplementary Fig. 2g), again suggesting SAPS3 is important for maintaining metabolic homeostasis under HFD but not required when the metabolic environment is balanced. LKO mice also showed increased levels of the plasma C-peptide, a proportional readout for insulin sensitivity (Fig. 3i). Next, we investigated whether the function of SAPS3 is through regulating AMPK activation and found that SAPS3 deletion led to an increase in phospho-AMPK and phospho-ACC compared to FF under HFD (Fig. 3j and Supplementary Fig. 2h). Therefore, liver-specific SAPS3 knockout recapitulates the phenotype observed in SAPS3 whole body knock-out and is sufficient to hinder the onset of diet-induced liver steatosis, obesity, and insulin resistance.

### Loss of SAPS3 reverses metabolic and transcriptional alterations induced by HFD

**Fig. 4:**
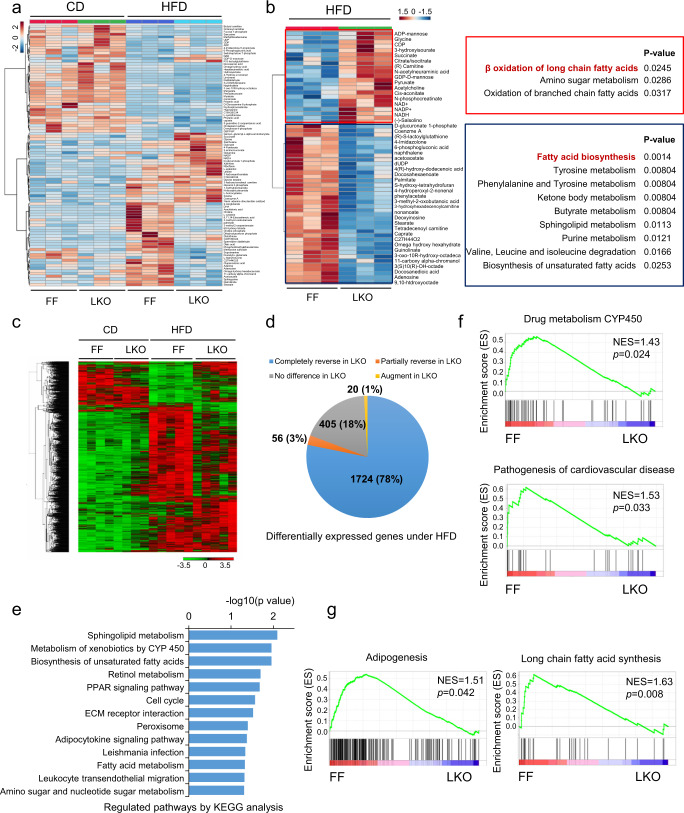
Loss of SAPS3 reverses metabolic and transcriptional alterations induced by HFD. **a** Heatmap of significantly changed metabolites in liver samples from FF and LKO mice fed with different diets was analyzed by LC-MS. *n* = 3 mice per group analyzed by one-way ANOVA. **b** Pathway analysis of upregulated metabolites and downregulated metabolites from FF and LKO mice respectively. *n* = 3 mice per group analyzed by unpaired *t*-test. **c** Transcriptional profile heatmap denoting significantly changed genes in liver samples from FF and LKO mice fed with different diets. CD *n* = 4 mice per group; HFD, *n* = 5 per group. Fold change > 1.5, *p* < 0.05 and FDR < 0.05. **d** Distribution in genes that were changed in FF mice by HFD and completely reversed (78%), partially reversed (3%), no difference (18%), or augmented (1%) by the knockout of SAPS3. **e** KEGG pathway analysis of the significantly reversed genes by SAPS3 knockout. CD *n* = 4 mice per group; HFD, *n* = 5 per group. Fold change > 1.5, *p* < 0.05, and FDR < 0.05. **f**, **g** GSEA analysis of the genes from FF and LKO mice under HFD. Adipogenesis and long-chain fatty acid synthesis were identified and shown in **g**.

To systemically examine the effects of SAPS3 deletion on liver metabolism, we performed metabolic profile analysis using liver samples from both FF and LKO mice fed with control and high-fat diets. Interestingly, SAPS3 knockout did not exhibit much effect on the metabolic landscape under a control diet further supporting the phenotypic data reported above. In contrast, HFD induced significant changes to metabolite pools in WT mice and most of the metabolites increased were reversed in LKO mice (Fig. 4a and Supplementary Data 1). We next used unbiased KEGG pathway analysis to understand the effect of HFD on metabolic alterations and observed many metabolic pathways are affected by HFD (Supplementary Fig. 3a). Among those altered pathways, we found most of them are impacted by the loss of SAPS3 (Supplementary Fig. 3b). To further determine the effects of SAPS3 deletion on metabolic regulations, we distinguished upregulated metabolites and downregulated metabolites in SAPS3 LKO mice compared to FF mice. Interestingly, we found that fatty acid oxidation was significantly upregulated in LKO mice, while fatty acid biosynthesis was the most downregulated pathway in LKO mice, suggesting loss of SAPS3 reverses the fatty acid metabolism altered by HFD in the liver (Fig. 4b and Supplementary Fig. 3c). Notably, LKO mice exhibited more evidence of fatty acid breakdown than synthesis compared to FF under HFD. We observed a significantly higher concentration of the FAO-related metabolites, including coenzyme A, carnitine, and NAD^+^, in LKO mice compared to FF under HFD (Supplementary Fig. 3d). Moreover, pools of palmitoylcarnitine, the direct product of the FAO initializing enzyme carnitine palmitoyltransferase 1 (CPT1) were increased further suggesting FAO is increased in the livers of these mice (Supplementary Fig. 3d). In contrast, the long-chain fatty acids, palmitate, stearate, and oleate were reduced in LKO mice under HFD, showing a lower level of fatty acid synthesis (Supplementary Fig. 3e). Moreover, it has been reported that HFD leads to decreased glucose uptake and glycolysis^22^. Consistently, we found decreased glycolytic intermediates upon HFD in FF mice, such as pyruvate and phosphoenolpyruvate, which can be reversed in LKO mice (Supplementary Fig. 3f). AMP is an important regulator of AMPK, therefore, we further compared the AMP levels between WT and KO samples and found no significant difference in AMP levels between WT and KO cells, indicating regulation of AMPK activity in SAPS3 KO cells is independent of AMP concentrations (Supplementary Fig. 3g). Thus, loss of SAPS3 causes a metabolic landscape in the liver that mirrors increased fatty acid oxidation and decreased fatty acid synthesis.

Metabolic syndromes bring about diverse and severe changes to the transcriptional landscape. Indeed, HFD is reported to induce changes in gene expression profiles. With this in mind, we investigated the gene expression profile of the livers from mice lacking SAPS3 and fed HFD. We performed RNA-seq from liver tissues collected from FF and LKO mice following 16 weeks on HFD. The liver samples from FF and LKO mice under CD or HFD clustered by genotype, confirming that genotype-dependent transcriptional changes dominated the dataset. Interestingly, there was almost no difference in gene expression between FF and LKO livers under CD, again enforcing the idea that SAPS3 may be a metabolic stress-responsive protein. In contrast, HFD-induced differential gene expression is significant in SAPS3 knockout mice (Fig. 4c). Among 2205 genes that were significantly altered by HFD in FF mice, 1724 (78%) of these genes were completely reversed by the deletion of SAPS3, and 56 (3%) of these genes were partially reversed by SAPS3 knockout (Fig. 4d and Supplementary Fig. 4a). Together, we identified 1780 (81%) out of 2205 genes that were altered by HFD but reversed by SAPS3 deletion, suggesting that SAPS3 deletion hinders the transcriptional changes caused by HFD. To better understand the networks of genes regulated by SAPS3 during HFD, we used unbiased KEGG pathway analysis on the set of 1780 genes and found the most affected pathways by loss of SAPS3 were metabolic pathways that involve lipid handling, including Sphingolipid metabolism, biosynthesis of unsaturated fatty acids and fatty acid metabolism (Fig. 4e). Additionally, the PPAR pathway and adipocytokine pathway, which are activated by HFD^23,24^, were decreased by SAPS3 knockout. Moreover, we used gene set enrichment analysis (GSEA) to unbiasedly explore the differential pathways in FF and LKO mice fed HFD. We found that CYP450 metabolism, the reported top-upregulated pathway in mice in response to HFD^25^, was reversed by the deletion of SAPS3 (Fig. 4f). One of the most well-known consequences of HFD is cardiovascular diseases. Remarkably, the pathway involving pathogenesis of cardiovascular diseases was decreased by SAPS3 knockout (Fig. 4f). Importantly, and in line with our metabolomics analysis, adipogenesis and long-chain fatty acid synthesis were impaired by the loss of SAPS3 (Fig. 4g). When we investigated the expression of the gatekeeper enzymes for fatty acid synthase in the LKO and FF mice following HFD, we found decreased expression of fatty acid synthase (FAS) and acetyl-CoA carboxylase 2 (ACC2) further supporting the loss of SAPS3 blocks lipogenesis in the liver (Supplementary Fig. 4b). To further confirm the fatty acid synthesis was inhibited by the loss of SAPS3, we performed experiments using stable isotope-labeling. Using labeled palmitate tracing in WT and KO MEF cells, we found less stearate and oleate were synthesized from U-^13^C_16_ palmitate when SAPS3 was lost, indicating synthesis of both saturated and unsaturated fatty acids is affected by SAPS3 deletion (Supplementary Fig. 4c). Thus, RNA expression profiling data demonstrate that the deletion of SAPS3 has a significant effect on gene expression under HFD and a limited influence on gene expression during balanced nutrition.

### SAPS3 regulates cellular metabolism via AMPK

**Fig. 5:**
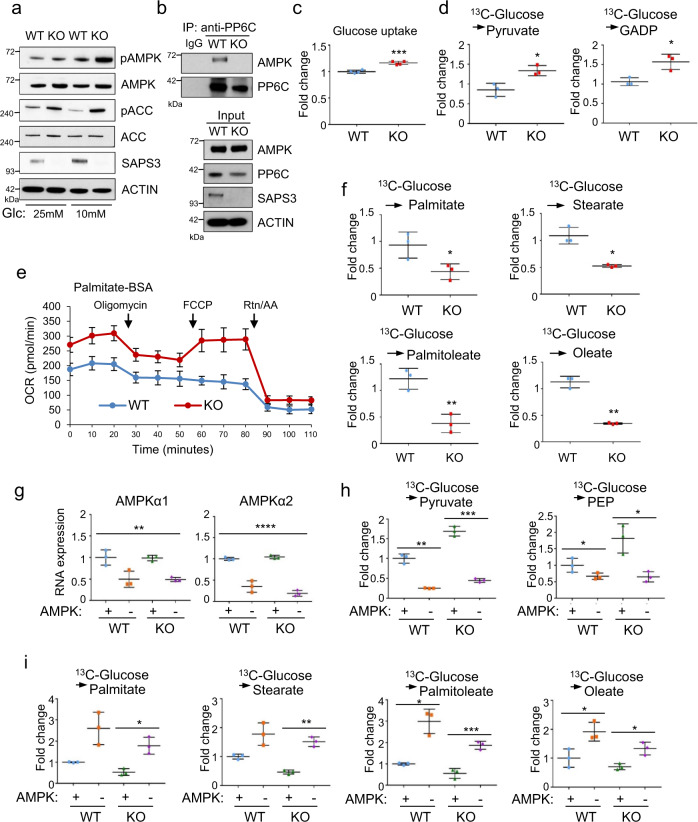
SAPS3 regulates cellular metabolism via AMPK. **a** pAMPK and downstream pACC were tested by Western blotting in WT and SAPS3 KO MEF cultured in 25 mM and 10 mM glucose medium. The results are representative of three independent experiments. **b** WT and SAPS3 KO MEF cell lysates were immunoprecipitated with anti-PP6C antibody followed by Western blotting. The results are representative of three independent experiments. **c** Glucose uptake was measured using the Nova Bioprofile 100 analyzer. Mean ± s.d., *n* = 4 biological replicates analyzed by two-tailed *t*-test, ***p = 0.0006. **d** U-^13^C_6_ glucose- derived pyruvate and glyceraldehyde-3-phosphate (GADP) levels in WT and SAPS3 KO MEF cells. Mean ± s.d., *n* = 3 biological replicates analyzed by two-tailed *t*-test. Pyruvate, *p = 0.018; GADP, *p = 0.028. **e** Fatty acid oxidation rate was measured using the XF-24 Seahorse system. Mean ± s.d., *n* = 5 biological replicates. **f** U-^13^C_6_ glucose-derived palmitate, stearate, palmitoleate, and oleate levels in WT and SAPS3 KO MEF cells. Mean ± s.d., *n* = 3 biological replicates analyzed by two-tailed *t*-test. Palmiate, *p = 0.049; Stearate, *p = 0.022; Palmitoleate, **p = 0.005; Oleate, **p = 0.005. **g** AMPKα1 and α2 expression in Crispr AMPK knockout MEF cells. Mean ± s.d., *n* = 3 biological replicates analyzed by one-way ANOVA, **p = 0.0015; ****p < 0.001. **h** U-^13^C_6_ glucose-derived pyruvate and phosphoenolpyruvate (PEP) levels in WT and SAPS3 KO MEF cells with or without AMPK. Mean ± s.d., *n* = 3 biological replicates analyzed by two-tailed *t*-test. Pyruvate, **p = 0.008; ***p = 0.001; PEP, WT, *p = 0.039; KO, *p = 0.032. **i** U-^13^C_6_ glucose derived palmitate, stearate, palmitoleate, and oleate levels in WT and SAPS3 KO MEF cells with or without AMPK. Mean ± s.d., *n* = 3 biological replicates analyzed by two-tailed *t*-test. Palmitate, *p = 0.02; Stearate, **p = 0.003; Palmitoleate, *p = 0.025, ***p = 0.001; Oleate, WT, *p = 0.025; KO, *p = 0.024.

Consistent with in vivo results, we found knockout of SAPS3 in MEFs leads to significantly increased phospho-AMPK and phospho-ACC in 25 mM and 10 mM glucose culture (Fig. 5a and Supplementary Fig. 5a). Moreover, we were not able to detect PP6C in complex with AMPK in these SAPS3 knockout cells (Fig. 5b). As AMPK has been implicated in the regulation of a number of metabolic pathways, including activation of glycolysis and fatty acid oxidation (FAO)^21,26,27^, and inhibition of fatty acid synthesis (FASN)^28^, we asked whether SAPS3 affects AMPK mediated metabolic functions. We found glucose uptake was significantly increased in SAPS3 KO MEFs (Fig. 5c). To better understand how glucose in cells was being stored in the absence of SAPS3, we used ^13^C-glucose stable isotope tracer-labeling and identified metabolites for incorporation of heavy glucose. Consistently, the key labeled glycolytic intermediates were increased when SAPS3 is deleted in cells (Fig. 5d). To examine whether increased glucose consumption is channeled to produce lactate or into the TCA cycle, we measured the lactate and TCA intermediates from labeled glucose. The data showed that loss of SAPS3 led to the increase in both lactate and TCA intermediates, suggesting both pathways increase their consumption of glucose carbon (Supplementary Fig. 5b). Using an oxygen consumption rate (OCR) based palmitate oxidation stress assay, a metric of long-chain fatty acid oxidation, we found FAO was significantly increased in SAPS3 KO MEFs (Fig. 5e). Moreover, these cells also displayed decreased levels of labeled palmitate, stearate, oleate, and palmitoleate (Fig. 5f), indicating inhibition of de novo lipogenesis in SAPS3 KO MEFs compared to WT MEFs.

To further confirm if SAPS3 deletion regulates metabolic pathways through activation of AMPK, we knocked out AMPK α1 and α2 in WT and SAPS3 KO MEF cells using CRISPR- based gene editing (Fig. 5g). As above, we used ^13^C-glucose isotope tracer labeling to follow the fate of glucose to fatty acids. Deletion of AMPK significantly reduced glycolytic intermediates in WT cells as previously shown^29^. As expected, the knockout of SAPS3 increased the concentration of glycolytic intermediates, which can be completely blocked by the deletion of AMPK (Fig. 5h). Meanwhile, loss of AMPK reversed the levels of labeled fatty acids that were reduced by SAPS3 knockout (Fig. 5i). Taken together, these results demonstrate that metabolic alterations in SAPS3 knockout cells were largely through activation of AMPK.

### SAPS3 deficiency-mediated metabolic homeostasis in vivo under HFD is AMPK dependent

**Fig. 6:**
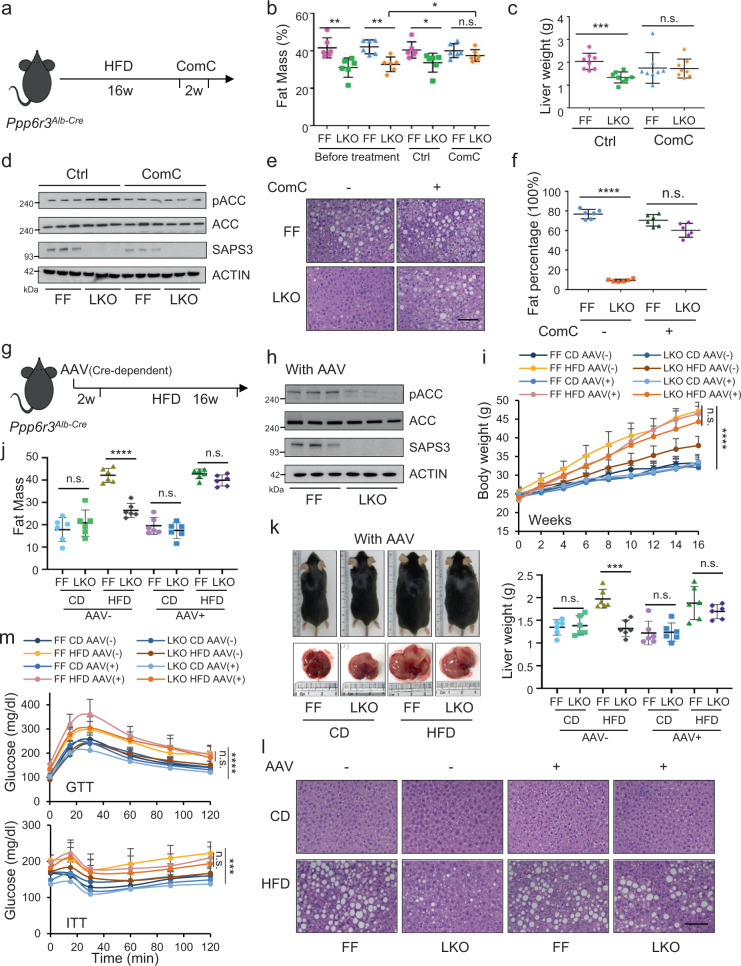
SAPS3 deficiency-mediated metabolic homeostasis in vivo under HFD is AMPK dependent. **a** Experimental strategy for feeding 8 weeks old male LKO mice with HFD (45 kcal% fat) for 16 weeks and then treating mice with compound C for two weeks. **b** Fat mass composition of FF and LKO male mice under HFD before compound C treatment and after compound C treatment for two weeks. Mean ± s.d., *n* = 6 mice per group analyzed by two-tailed *t*-test. Before treatment, FF/LKO, **p = 0.006, **p = 0.0017; After treatment, FF/LKO, *p = 0.033; n.s., not significant.; LKO, before treatment/comC, *p = 0.048. **c** Liver weights from mice treated with ctrl or compound C. Mean ± s.d., *n* = 8 mice per group analyzed by two-tailed *t*-test, ***p = 0.0005; n.s., not significant. **d** AMPK mediated ACC phosphorylation was tested by Western blotting. The results are representative of two independent experiments. **e** Representative images of H&E staining of liver slides from FF and LKO mice. Scale bar, 100 µm. **f** The quantification of the H&E staining images shown in **e**. Mean ± s.d., *n* = 6 mice per group analyzed by two-tailed *t*-test, ****p = 8.7918E-08; n.s., not significant. **g** Experimental strategy for injecting AAV-AMPK-DN in 6 weeks old male LKO mice and, after two weeks, followed by feeding mice with HFD (45 kcal% fat) for 16 weeks. **h** The inhibition of AMPK activity by AAV-AMPK-DN was evaluated by the level of phospho-ACC from liver samples after HFD. The results are representative of two independent experiments. **i** Body weight was measured. Mean ± s.d., *n* = 6 mice per group analyzed by two-tailed *t*-test, n.s., not significant; or one-way ANOVA, ****p < 0.0001. **j** Fat compositions were measured by echo-MRI. Mean ± s.d., *n* = 6 mice per group analyzed by two-tailed *t*-test, ****p = 5.9683E-06; n.s., not significant. **k** Representative pictures of mice and livers from FF and LKO mice. And the liver weights were compared. Mean ± s.d., *n* = 6 mice per group analyzed by two-tailed *t*-test, ***p = 0.00018; n.s., not significant. **l** Representative images of H&E staining of liver slides from FF and LKO mice with or without AAV. Scale bar, 100 µm. CD, AAV (−) *n* = 3 mice per group; CD, AAV ( + ) *n* = 4 mice per group; HFD, AAV (−) *n* = 6 mice per group; HFD, AAV ( + ) *n* = 6 mice per group. **m** Glucose tolerance test and insulin tolerance test were performed at the end of the experiment. Mean ± s.d., *n* = 6 per group analyzed by one-way ANOVA or two-tailed *t*-test. ****p < 0.0001; ***p = 0.0001; n.s., not significant.

To investigate whether the metabolic effect induced by SAPS3 deletion is via activation of AMPK in vivo, we treated LKO mice with Compound C, a non-selective AMPK inhibitor, after 16 weeks on HFD (Fig. 6a). As observed before, the HFD-induced increases in fat mass and liver weights observed in FF mice were significantly reduced in SAPS3 LKO mice. However, treatment with Compound C largely diminished the effect of SAPS3 deletion on fat mass and liver weights (Fig. 6b, c). In addition, reduced phosphorylation of the canonical AMPK substrate ACC was found in both FF and LKO livers upon compound C treatment, confirming the deactivation of AMPK by Compound C (Fig. 6d and Supplementary Fig. 6a). Importantly, Compound C substantially increased the lipid droplets in SAPS3 LKO liver samples compared to control-treated mice (Fig. 6e,f). We found normal levels of alanine transaminase (ALT) and aspartate transaminase (AST) in mice livers excluding the possibility that Compound C impairs liver functions (Supplementary Fig. 6b). These data suggest the inhibition of AMPK largely reverses the phenotype in SAPS3 knockout mice and further demonstrate these two work together to modulate the response to HFD in vivo.

To further determine whether SAPS3 functions directly through hepatic activation of AMPK, we used Cre-dependent adeno-associated virus (AAV) expressing a dominant negative (DN) kinase dead (K45R) form of AMPK in the liver^30^, then fed with HFD (Fig. 6g). Since LKO mice have liver-specific expression of Cre protein, AMPK-DN was only expressed in LKO mice, not in FF mice. The AMPK-DN expression was confirmed via Western blotting by reduced phospho-ACC in liver samples after HFD (Fig. 6h and Supplementary Fig. 6c). As a control, we found there was no effect on phospho-ACC in lung tissue (Supplementary Fig. 6d). We found the body weights of LKO mice with AAV-AMPK-DN were similar to FF mice under HFD (Fig. 6i) with similar food intakes (Supplementary Fig. 6e), suggesting that AMPK-DN suppresses the beneficial effects found in SAPS3 LKO mice in response to HFD. Moreover, AMPK-DN reverses the effect of SAPS3 knock-out on fatty acid synthesis and lipid accumulation in vivo (Fig. 6j). We also observed a similar level of liver hypertrophy and liver steatosis in LKO mice treated with AAV-AMPK-DN compared to FF mice (Fig. 6k, l and Supplementary Fig. 6f). When we investigated glucose and insulin sensitivity in these mice, we found under HFD, LKO mice with AMPK-DN reversed the sensitivity exhibited by SAPS3 LKO (Fig. 6m and Supplementary Fig. 6g) and also reversed the levels of fasting blood glucose (Supplementary Fig. 6h).

## Discussion

It has been well studied that AMPK activity is tightly and dynamically regulated by reversible protein phosphorylation^31,32^. However, the specific protein phosphatase complex that can directly dephosphorylate AMPK and shut down AMPK-mediated metabolic responses is still unknown. Despite studies that have shown many catalytic subunits of serine-threonine protein phosphatases, including protein phosphatase 2 C and protein phosphatase 2 A, can dephosphorylate AMPK in vitro, they may not function as an AMPK phosphatase in vivo since catalytic subunits of serine-threonine protein phosphatase are constitutively active with promiscuous activity towards substrates^6,33^. The substrate specificity of the serine-threonine protein phosphatase is largely determined by the regulatory subunits of serine-threonine protein phosphatases. Here, we found that protein phosphatase 6 regulatory subunit, SAPS3, was the top binding protein to AMPK and functions as a central regulator of metabolism. Deletion of SAPS3 in mice leads to AMPK activation and protects against the HFD-induced detrimental metabolic phenotypes by improving glucose homeostasis and insulin sensitivity. Interestingly, *PPP6R3*, the gene that encodes SAPS3, is located on the type 1 diabetes susceptibility locus, IDDM4, on chromosome 11q13^34^. SAPS3 was also reported as being among obesity or diabetes-related genes in the human gene database *GeneHancer* and there is a significant association of higher expression level of SAPS3 with T2D based on sequencing data and genome-wide association studies (GWAS)^35–37^. A recent report indicates that in yeast, PP6 family phosphatase Ppe1 is the primary phosphatase for Ssp2/AMPKα dephosphorylation, suggesting a conserved role for the PP6 phosphatase complex in AMPK dephosphorylation and links SAPS3 to the genesis of nutrient-sensing^38^. Recent data indicate that AMPK plays a global role in regulating aspects of whole-body energy balance including appetite and body weight. Metabolically, AMPK is well known to be a major mediator of glucose and lipid homeostasis^39–41^ and activation of hepatic AMPK is essential for whole-body metabolism, such as control of blood glucose levels and plasma triglyceride levels^42–44^. Consistent with this, we found that liver-specific deletion of SAPS3 is sufficient to affect whole-body metabolism under a high-fat diet. These results support the notion that activation of the AMPK pathway in the liver is sufficient to regulate whole-body metabolism. Interestingly, we found no significant difference in food intake between WT and SAPS3 KO mice under HFD. Besides food intake, the resistance to body weight increase might be caused by the impact of SAPS3 deletion on energy expenditure since recent publications indicate that AMPK plays a role in energy expenditure^45,46^.

Interestingly, we found that deletion of SAPS3 in mice had minimal effects on mice development and biological functions during balanced nutritional intake. Consistently, the metabolomic and genomic analysis also confirmed that SAPS3 deletion issued no significant changes on metabolism and transcription under the control diet. These data suggest that SAPS3 mainly functions under metabolic stress conditions, induced by high-fat diet, which are critical causes for metabolic perturbations. Thus, SAPS3 deficiency functions as a guardian to maintain metabolic homeostasis upon stress conditions. We also noticed there are increased levels of many metabolites in LKO mice under HFD compared to mice under control diet. After statistical analysis by Metaboanalyst, three pathways were shown to be affected by the knockout of SAPS3 under HFD compared to control diet: glycine metabolism, citric acid cycle, and β oxidation of long-chain fatty acids (Supplementary Fig. 2b). Glycine and serine metabolism are related to glutathione synthesis that protects and assists fatty acid oxidation^47^, the TCA is also a source for fatty acid oxidation^48^. Therefore, the three pathways might cooperatively boost the reduction of fatty acids in vivo.

AMPK is known to be a major mediator of energy homeostasis. By regulating tissues such as liver, muscle, and adipose tissues, AMPK controls whole-body glucose homeostasis^41^. Meanwhile, lipid synthesis and oxidation in the liver, and lipolysis and lipogenesis in adipose tissue are also tightly regulated by AMPK. Thus, using genetic expression of activated AMPK in the liver could lower blood glucose levels and improve lipid profile^49,50^. As a result, many different AMPK activators were developed to control blood glucose levels and plasma triglyceride levels in metabolic syndromes^51,52^. However, the role of hepatic AMPK in regulating blood glucose levels is still controversial. Studies using liver-specific AMPK knockout mouse models or pharmaceutical AMPK activators that target hepatic AMPK failed to show significant impact on blood glucose levels^53,54^. On the other hand, genetic expression of activated AMPK in liver is sufficient to lower blood glucose levels and improve lipid profile^49,50^. Consistently, our data show that liver-specific deletion of SAPS3, which leads to AMPK activation, is sufficient to improve blood glucose levels under HFD.

In summary, our data provide a more complete understanding of the biological response to unbalanced nutrition. We identify SAPS3 containing PP6 complex as an AMPK phosphatase that plays a central role in the response to metabolic perturbations upon dietary challenges. Thus, inhibition of SAPS3 function provides a strong potential for the development of novel AMPK activators to treat metabolic syndromes.

## Methods

### Mice and ethics statement

All studies involving mice were performed according to the Institutional Animal Care and Use Committee (IACUC)-approved protocols at the University of California, Irvine (IACUC protocol: AUP-20-159). We have complied with the relevant ethical considerations for animal research overseen by this committee. *Ppp6r3*^*fl/fl*^ C57BL/6 mice were generated by inGenious Targeting Laboratory. *Ppp6r3*^*+/-*^ mice were generated by microinjecting Cre recombinase RNA to *ppp6r3*^*wt/fl*^ embryos^55^. WT and SAPS3 KO mice were generated by breeding *ppp6r3*^*+/-*^ mice. LKO mice were generated by breeding *ppp6r3*^*fl/fl*^ mice with C57BL/6^Alb-Cre^ mice obtained from the Jackson Laboratory. The following diets were used: Control diet (10 kcal% fat, D12450B, Research Diet Inc.); High Fat Diet (45 kcal% fat, D12451, and 32.5 kcal% fat, D12266, Research Diet Inc.). All mice were housed on a 12 h light/12 h dark cycle with free access to water and diet. Body weights were measured every week. Body fat and lean mass were measured in conscious animals using a whole-body composition analyzer (EchoMRI). For Compound C treatment, 3 mg/kg of compound C was intraperitoneally injected into the experimental group mice every day for two weeks. For AAV injection, pAAV-EF1a-Flex-DN-AMPK-K45R-T2A-mCherry plasmid^56^ was amplified in recombination-deficient bacteria Stbl3, and Serotype 8 AAVs were packaged by the University of North Carolina Vector Core and the following titer was achieved (vg/ ml): 1.9 × 10^12^.

### Cell lines and cell culture

293 T cells (ATCC® CRL-3216™) and HT1080 cells (ATCC® CCL-121™) were purchased from American Type Culture Collection (ATCC). SAPS3 KO and WT MEFs were generated from SAPS3 KO and WT mice. All cells were cultured in Dulbecco’s modified Eagle’s medium (DMEM) (10-017-CV, Corning) supplemented with 10% fetal bovine serum (FBS) (100−106, Gemini Bio-Products), 100 units/mL of penicillin, and 100 μg/mL of streptomycin (516106, Sigma). For glucose deprivation and add-back treatment, HT1080 cells were cultured in DMEM without glucose (11966025, Gibco), supplemented with 10% dialyzed FBS (FB-07, Omega) for 1 h, then the medium was replaced with the complete medium for 20 min. 293 T cells were cultured in DMEM without glucose, supplemented with 10% dialyzed FBS for 6 h, then the medium was replaced with the complete medium for 1 h or 3 h. For siRNA transfection, siSAPS1 (L-020420-01-0005, Horizon), siSAPS2 (L-021331-01-0005, Horizon) and siSAPS3 (L-014646-01-0005, Horizon) were transfected by Lipofectamine RNAiMAX transfection reagent (13778150, Invitrogen). To generate lentiviral particles, 293 T cells were cultured in a 6 cm dish and co-transfected with 1 μg control or shRNA vector, 0.5 μg pCMV-VSV-G, and 1 μg pCMV-dR8.2 dvpr by Lipofectamine 2000 (11668−019, Invitrogen) transfection reagent. The viral supernatants were collected and filtered. Cells were infected with the virus with 10 μg/ml polybrene.

### Protein mass spectrometric peptide sequencing

A total of 293 T cells were transfected with plasmids encoding Flag-tagged AMPKα2 protein. Cells were deprived of glucose for 6 h and glucose was added back for 1 h, followed by lysis using RIPA buffer (150 mM KCl, 0.2% (v/v) NP-40, 10% (v/v) glycerol, 20 mM Tris at pH 7.5, 0.5 mM DTT) containing protease inhibitor complex (4693159001, Roche) and phosphatase inhibitor (1862495, Thermo fisher scientific). 20 μl anti-Flag beads (A2220, Sigma) were added to the cell lysates and rotated overnight. Beads were washed four times with cold lysis buffer and eluted with Flag peptide (F3290, Sigma) for 4 h. The elutes were separated by SDS-PAGE and visualized by Coomassie Blue staining (LC6025, Invitrogen). The binding proteins were identified by mass spectrometry analysis, performed by the Taplin Mass Spectrometry Facility at Harvard Medical School.

### Immunofluorescence (IF)

Cells were washed twice with PBS and fixed with 4% formaldehyde (Thermo Scientific, 28908) in PBS for 15 min at room temperature. Cells were washed again three times with PBS and permeabilized with 0.1% TritonX-100 (Fisher, BP151-500) in PBS at room temperature for 10 min. Then cells were washed three times with PBS and blocked with 3% BSA in PBS for 1 h. Thereafter, cells were incubated with anti-AMPKα (Thermo Scientific, MA537501, 1:50) and anti-SAPS3 (Thermo Scientific, 16944-1-AP, 1:100) antibodies overnight at 4 °C. Cells were washed three times with PBS and incubated with goat anti-rabbit 594 (Invitrogen, A11037, 1:500) and goat anti-mouse 488 (Invitrogen, A11029, 1:500) antibodies for 1 h at room temperature. After that, cells were washed three times and mounted with the Prolong Gold antifade reagent with DAPI (Invitrogen, P36931). The slides were visualized with Zeiss LSM 900 Airyscan.

### Immunoprecipitation (IP)

IP was performed as previously described^57^. Cells were cultured in 10 cm dishes in DMEM supplemented with 10% FBS. 80% confluent cells were washed twice with ice-cold PBS and lysed on ice using a cell scraper with lysis buffer (150 mM KCl, 0.2% (v/v) NP-40, 10% (v/v) glycerol, 20 mM Tris at pH 7.5, 0.5 mM DTT) containing protease inhibitor complex (4693159001, Roche). The protein concentrations of lysates were measured using the BCA Assay kit (23225, Thermo fisher scientific). 1 mg lysates were immunoprecipitated when rotating at 4֯C with 4 μg of antibodies or control IgG. A total of 20 μl Protein G agarose beads (15920010, Thermo fisher scientific), anti-Flag beads (A2220, Sigma), or anti-Myc beads (20169, Thermo fisher scientific) were added to each tube and rotated overnight. Beads were washed four times with cold lysis buffer, resuspended in SDS loading dye, and boiled for 5 min.

### Immunoblotting

Tissues were homogenized, or cells grown in 6 cm dish were lysed on ice in lysis buffer (50 mM Tris-HCL [pH 7.4], 5 mM Sodium Fluoride, 5 mM Sodium Pyrophosphate, 1 mM EDTA, 1 mM EGTA, 250 mM Mannitol, 1% [v/v] Triton X-100) containing protease inhibitor complex (04693159001, Roche) and phosphatase inhibitor (1862495, Thermo fisher scientific). The protein concentrations were measured by using the BCA Assay kit (23225, Thermo fisher scientific). The lysates were boiled with NuPAGE LDS-PAGE sample buffer (1771559, Invitrogen) supplemented with 5% β-mercaptoethanol (M3148, Sigma) for 5 min. Equal amounts of protein were loaded on precast NuPAGE Bis-Tris Gels (NP0321BOX, Life Technologies) followed by transfer onto nitrocellulose membrane (1620115, Bio-Rad). The immunoblotting was performed with the following antibodies: anti-AMPKα (ab80039, Abcam, 1:1000), anti-pAMPKα (ab133448, Abcam, 1:1000), anti-pAMPK (4188, Cell signaling, 1:1000), anti-ACC (3662, Cell signaling, 1:1000), anti-pACC (3661, Cell signaling, 1:1000), anti-β-ACTIN (A1978, Sigma,1:5000), anti-GAPDH (2218, Cell signaling, 1:1000), anti-SAPS3 (A300-971A, Bethyl, 1:1000), anti-PP6C (A300-844A, Bethyl, 1:1000), anti-MYC (ab9106, abcam, 1:1000), anti-FLAG (F3165, Sigma, 1:1000), anti-SAPS3 (A300-972A, Bethyl, 1:1000), anti-SAPS1 (A300-968A, Bethyl, 1:1000), and anti-SAPS2 (A300-969A, Bethyl, 1:1000). All the uncropped images of blot results were shown in Supplementary Fig.7.

### PCR and DNA gel electrophoresis

PCR was performed using GoTaq® Master Mixes (M7122, Promega). PCR products were detected using agarose gel electrophoresis (1.5%). PCR primers are *ppp6r3*-F: 5’-TTCACACATACCCAGGAATCAGA-3’; R: 5’-GACTGCTGAGACACAAGGGC-3’. The PCR cycle parameters were as follows: 95 ˚C for 3 min; 40 cycles with denaturation at 95 ˚C for 10 sec, annealing at 50 ˚C for 30 sec.

### Glucose tolerance and insulin tolerance

Blood glucose levels were determined using an automated blood glucose reader (Accu-Check; Roche). Glucose tolerance tests were performed on mice that were fasted overnight. Blood was collected immediately before as well as 15, 30, 60, 90, and 120 min after intraperitoneal injection of glucose (1 g/kg body weight). For insulin tolerance tests, mice were fasted for 3 h and then injected with 0.5 U/kg body weight of insulin (91077 C, Sigma). Blood glucose was measured at 0, 15, 30, 60, 90, and 120 min.

### Haematoxylin and eosin (H&E) staining

After mice were euthanized, tissues were collected and fixed in 10% formalin. Formalin-fixed, paraffin-embedded blocks of tissues were used for haematoxylin and eosin (H&E) staining^57^. 5 µm paraffin-embedded formalin-fixed slides were deparaffinized with xylene, and rehydrated by different concentrations of ethanol. Rinse the slides in distilled water and stain them in hematoxylin/Eosin. After dehydrating the slides in different concentrations of ethanol, slides were cleared in Xylene, and mounted with Cytoseal (Thermo fisher scientific). Image analysis was performed under a microscope (SeBa Laxco).

### Serum biochemistry

Mice were fasted for 4 h before blood was collected via cardiac puncture in BD vacutainer tubes (Fisher Scientific). The serum was obtained by centrifugation at 2000 x *g* for 15 min. Serum triglyceride (ab65336, Abcam), C-peptide (90050, Crystal Chem), alanine transaminase (ALT) (EALT-100, EnzyChrom), and aspartate transaminase (AST) (EASTR-100, BioAssay System) were measured according to manufacturers’ instructions.

For the triglyceride test, 50 µl standard dilutions or samples (5 µl sample was adjusted volume to 50 µl with triglyceride assay buffer) were loaded into plates. Then, 50 µl of the reaction mixture was added to each standard, sample, and background control wells. The plate was mixed and incubated at room temperature for 60 min protected from light. After that, the absorbance was measured using a plate reader.

For the C-peptide test, 95 µl of sample diluent and 5 µl of sample or working standards were added to each well. The microplate was covered with the plate sealer and mixed for 10 sec. Then, the plate was incubated for 1 h at room temperature. The contents were aspirated, and the wells were washed with 300 µl of wash buffer six times followed by adding 100 µl anti-C peptide enzyme conjugate. The microplate was covered with the plate sealer and incubated for 1 h at room temperature. After that, contents were aspirated, and wells were washed with 300 µl of wash buffer six times. Immediately, 100 µl per well of the enzyme-substrate solution was dispensed. The plate was covered to let the mix react for 30 min at room temperature. In the end, the reaction was stopped by adding 100 µl of enzyme reaction stop solution, and the absorbance was measured using a plate reader.

For the ALT test, 20 µl of each sample was transferred to a 96 well plate. 200 µl working reagent (200 µl assay buffer, 5 µl cosubstrate, 1 µl LDH, and 4 µl reconstituted NADH) were added to the sample and standard wells. The plate was taped to mix and incubated at 37  ֯C. The plate was read at 5 min and 10 min. For the AST test, 20 µl of each sample was transferred to a 96 well plate. 200 µl working reagent (200 µl assay buffer, 1 µl cofactor, 1 µl enzyme mix, and 4 µl NADH) were added to the sample and standard wells. The plate was taped to mix and incubated at 37  ֯C. The plate was read at 5 min and 10 min.

### Liquid chromatography-mass spectrometry (LC-MS)

A total of 3−10 mg of tissue samples were cut on dry ice and soaked in pre-cooled 80% methanol in HPLC-grade water. Samples were homogenized by Precellys 24 homogenizer using Precellys ceramic kit. After centrifuging at 17,000 x *g* at 4 ֯C for 10 min, the supernatant was transferred to a new tube and dried by speed vacuum. Liquid chromatography-mass spectrometry (LC-MS) was carried out at Duke University. The HPLC (Ultimate 3000 UHPLC) with an Xbridge amide column (Waters) is coupled to Q exactive plus hybrid quadrupole-orbitrap mass spectrometer (QE-MS) (Thermo Scientific) for compound separation and detection^58^. 301 metabolites were detected from all of the samples Mass isotopomer distributions were determined by integrating metabolite ion fragments and corrected for natural abundance as described^59^. MetaboAnalyst was used to analyze significantly changed metabolites and generate the heatmap and KEGG pathway analysis (www.metaboanalyst.ca/).

### Purified protein pull-down assay

The pull-down was performed as previously described^60^. 3 μg of recombinant Myc-PPP6R3 protein (TP328606, OriGene), GST-PPP6C protein (H00005537-P01, Novus), His-pAMPK (P48-10H-10, SignalChem) or His-AMPK (P48-14H-20, SignalChem) were mixed. The mixture was incubated at 37 ֯C for 1 h in 200 μl of assay buffer containing 25 mM Tris (pH8.0), 150 mM NaCl, 2 mM dithiothreitol, and 1 mg/ml BSA to block non-specific binding. Then, 20 μl His select Nickel affinity beads (P6611, Sigma) or 20 μl Protein G agarose beads with 4 μl anti-PP6C antibody were added to the assay buffer and rotated for 1 h at room temperature. After three washes with the assay buffer, the beads were resuspended in SDS loading dye, and examined by western blotting.

### Glucose uptake measurement

A total of 2 × 10^5^ cells WT or SAPS3 KO MEFs were plated on each well of a 6-well plate and cultured overnight. Then, the medium was replaced with 1 mL 10 mM glucose DMEM supplemented with 10% FBS. After culturing for 24 h, the medium was collected, and the cell number was counted by an automated cell counter (Bio-Rad). After cell debris was spun down, 0.8 mL medium was used to measure glucose by the Nova Biomedical BioProfile 100 with fresh 10 mM glucose DMEM as control. This analysis was as followed: glucose uptake = glucose in fresh medium (mM)-glucose in cultured medium (mM). All metabolite measurements were normalized based on cell number.

### ^13^C_6_-Glucose and ^13^C_16_-Palmitate tracing by GC-MS

2×10^5^ cells were seeded in 6 cm plates containing DMEM supplemented with 10% FBS and cultured overnight. Cells were washed with PBS twice and cultured in glucose-free DMEM supplemented with 10% dialyzed FBS containing ^13^C_6_-glucose (10 mM; Cambridge Isotope Laboratory). After culturing for 6 h which led to 85% labeling of the total glucose pool, the medium was aspirated at room temperature. Cells were washed by cold saline and put on dry ice. U-^13^C_16_-palmitate was complexed to fatty-acid-free BSA (A8806, Sigma) in 6:1 molar ratio in PBS by rolling overnight at room temperature. When cells were at 80% confluence, the medium was replaced by DMEM (A1443001, Gibco) with 10 mM glucose, 2 mM glutamine, 10% dialyzed FBS and 0.1 mM [U-^13^C_16_] palmitate. Cells were cultured in the labeled medium for 48 h which led to approximately 40% labeling of the total cellular palmitate pool.

For polar metabolites, 1 ml 80% methanol/water (HPLC grade) with norvaline as internal standard was added to cells. The plate was transferred to a −80 ˚C freezer and left for 15 min to further inactivate enzymes. Cells were then harvested by a silicone scraper and the whole-cell extract was transferred to a tube and centrifuged at 17000 x *g* for 10 min at 4 ˚C. The supernatant was transferred into tubes and dried by speed vacuum. Fifty microlitres of MOX (10 mg/ml in pyridine, 226904 Sigma) was added and the mixture was incubated at 42 ˚C for 1 h. After the samples were cooled down, 100 μl TBDMS (394882, Sigma) was added, and samples were incubated at 70 ˚C for 1 h. Then samples were transferred to GC vials and analyzed by Agilent 7820 A chromatography and Agilent 5977B mass spectrometer^57^.

For nonpolar metabolites, 500 μl methanol, 500 μl chloroform, 200 μl H_2_O and 10 μl 10 mM d31-C16:0 as internal standard was added to cells. The samples were vortexed and centrifuged at 14,000 xg for 5 min. The chloroform phase was dried and then derivatized to form fatty acid methyl esters (FAMEs) via addition of 500 μl 2% H_2_SO_4_ in methanol at 50 °C for 2 h followed by adding 100 μl saline and 500 μl hexane. After extraction, the hexane layer was dried and dissolved in 100 μl hexane. These samples were analyzed using a select FAME column.

### Seahorse assay for fatty acid oxidation

5×10^4^ cells were seeded into seahorse XF24 microplates (100850, Agilent) and cultured for 6 h. Before incubation, the culture medium was removed and cells were washed with substrate limited medium (DMEM supplemented with 0.5 mM Glucose, 1 mM GlutaMAX (35050061, Thermo fisher scientific), 0.5 mM Carnitine (C0283, Sigma), and 1% FBS). Then, 300 μl substrate limited medium was added to each well and cultured overnight. On the next day, the substrate limited medium was replaced by 375 µl FAO buffer (111 mM NaCl, 4.7 mM KCl, 1.25 mM CaCl_2_, 2 mM MgSO_4_, and 1.2 mM NaH_2_PO_4_) supplemented with 2.5 mM glucose, 0.5 mM carnitine, and 5 mM HEPES. After 30 min, 37.5 µl etomoxir (40 μM final) (236020, Sigma) was added to the FAO buffer. 15 min after that, 87.5 µl palmitate: BSA or BSA control was added into the buffer, and XF cell mito stress assay (103015-100, Agilent) was initiated. 56 µl oligomycin (30 µM), 62 µl FCCP (20 µM), and 69 µl Rotnone/Antimycin A (20 µM) were added to the cartridge wells. OCR level was determined using Seahorse bioscience XF24 extracellular flux analyzer (Agilent) and each cycle of measurement involved mixing (3 min), waiting (2 min), and measuring (3 min) cycles. The data were analyzed in Seahorse Wave Desktop Software 2.4.

### Crispr-cas9 knockout

Two oligos were designed to knockout AMPKα1 and α2 using the software at http://crispor.tefor.net/crispor.py. F-5’-CACCGGAAGCAGAAGCACGACGGGC-3’; R-5’-AAACGCCCGTCGTGCTTCTGCTTC. LentiCRISPRv2 encoding Cas9 (52961, Addgene) was digested with BsmBI (R0580, Biolabs) and purified by DNA gel. The two oligos were ligated to the digested plasmid. SAPS3 KO and WT MEFs were infected with lentivirus containing lentiCRISPRv2-AMPKα1/α2-specific oligos and then selected by puromycin.

### Quantitative real-time PCR

Total RNA was extracted using Trizol reagent. Reverse transcription reaction was performed using qScript cDNA Synthesis Kit (95047-100, Quanta Biosciences). qRT-PCR was performed in a CFX Connect Real-Time PCR Detection System (Bio-Rad) by using a reaction mixture with SYBR Green PCR Master Mix (95072, Quanta Biosciences). The PCR cycle parameters: 95 ˚C for 3 min; 40 cycles with denaturation at 95 ˚C for 10 sec, annealing at 55 ˚C for 30 sec. All the PCR amplification was performed in triplicate and repeated in three independent experiments. The relative quantities of genes were normalized to mouse 18 S RNA. The primers were as follows: *ppp6r1* F: 5’- TGATCGCTTCCATCAGCTCC −3’; *ppp6r1* R: 5’- GACCACGTGTAACCTCGTGT −3’; *ppp6r2* F: 5’- ATCCATCCCCACCAGGATGA −3’; *ppp6r2* R: 5’- CAGTCCTGCGACTCCAATGT −3’; ppp6r3 F: 5’- CTCCACAACCCAGGCAAGAT −3’; *ppp6r3* R: 5’- TGCCGATCTTCTTCTTGCGA −3’; *ampk α1* F: 5’- CGCAGACTCAGTTCCTGGAG −3’; *ampk α1* R: 5’- CTTCACTTTGCCGAAGGTGC −3’; *ampk α2* F: 5’- TCCTGAACACCTCAGCGTTC −3’; *ampk α2* R: 5’- CTTCCGGTCAAAGAGCCAGT −3’; *acc2* F: 5’- GAACCGGCTTCCTGGTTGTA −3’; *acc2* R: 5’- TCCTCCCCTATGCCGAAAGA −3’; *fas* F: 5’- CAAGTGTCCACCAACAAGCG −3’; *fas* R: 5’- GGAGCGCAGGATAGACTCAC −3’.

### Statistics

All experiments were repeated independently at least three times with similar results. Results are shown as means; error bars represent standard deviation (s.d.). The unpaired two-tailed Student’s *t*-test or one-way ANOVA was used to determine the statistical significance of differences between means (*p < 0.05, **p < 0.01, ***p < 0.001) and were calculated in Microsoft Excel or GraphPad Prism (v 9) software unless indicated separately.

### Reporting summary

Further information on research design is available in the Nature Portfolio Reporting Summary linked to this article.

## Supplementary information

Supplementary Information

Description of Additional Supplementary Files

Supplementary Data 1

Reporting Summary

## Data Availability

The RNA-seq data generated in this study have been deposited in the Gene Expression Omnibus (GEO) under the accession code: GSE210333. The mass spectrometry proteomics data generated in this study have been deposited to the ProteomeXchange Consortium via the Proteomics IDEntifications Database (PRIDE) partner repository under the accession code: PXD039625. The metabolomics data generated in this study are provided in the Supplementary Data 1. Source Data are provided with this paper, and the uncropped gel/blot images are provided in the Supplementary Figure 7. All other data sets are available within the article and supplementary information. Source data are provided with this paper.

## Source data

Source Data
