# Supplementary material for: SAPS3 subunit of protein phosphatase 6 is an AMPK inhibitor and controls metabolic homeostasis upon dietary challenge in male mice
Source: Nat Commun. 2023 Mar 13;14:1368. doi: 10.1038/s41467-023-36809-1 (PMC10011557; doi:10.1038/s41467-023-36809-1)

Supplementary Information

SAPS3 subunit of protein phosphatase 6 is an AMPK inhibitor and controls metabolic homeostasis upon dietary challenge in male mice

Yang Y et al.

Supplementary Fig.1 SAPS3 brings the PP6 catalytic subunit to dephosphorylate AMPK

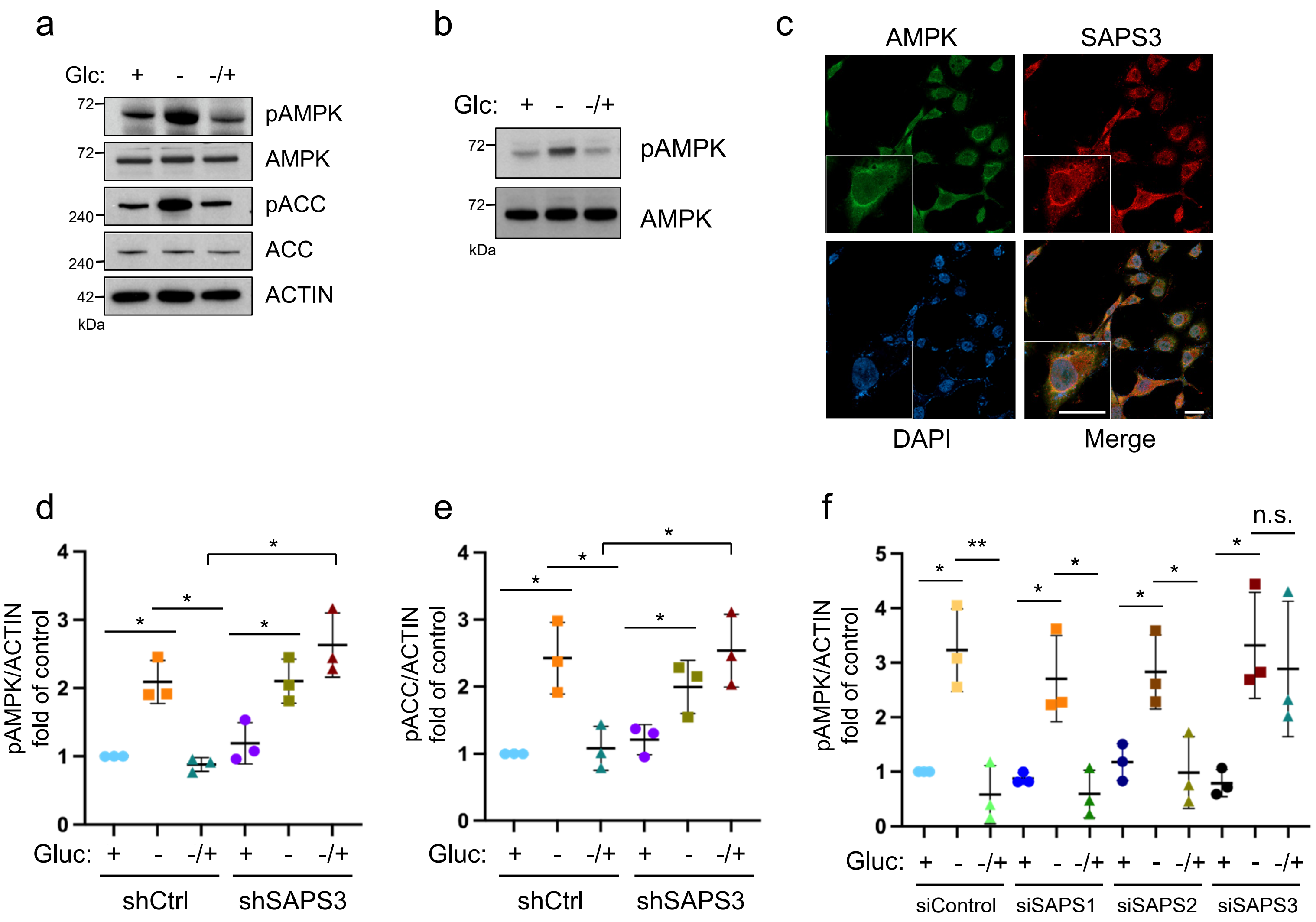

Supplementary Fig.1 SAPS3 brings the PP6 catalytic subunit to dephosphorylate AMPK.

a, b HT1080 and 293T cells were treated with glucose deprivation or add back (-/+). Cell lysates were analyzed by western blotting. The results are representative of three independent experiments. **c** HT1080 cells were immunostained with anti-SAPS3/anti-AMPKα and counterstained with DAPI. Scale bars, 20 μm. The results are representative of three independent experiments. **d, e,** and **f** Quantification of western blots Fig.1i and 1j. Mean ± s.d., n=3 biological replicates from three independent experiments analyzed by two-tailed t-test. d, shCtrl Gluc(+)/Gluc(-), **p*=0.026; shCtrl Gluc(-)/Gluc(-/+), **p*=0.014; shSAPS3 Gluc(+)/Gluc(-), **p*=0.015; Gluc(-/+) shCtrl/shSAPS3, **p*=0.015. e, shCtrl Gluc(+)/Gluc(-), **p*=0.043; shCtrl Gluc(-)/Gluc(-/+), **p*=0.028; shSAPS3 Gluc(+)/Gluc(-), **p*=0.036; Gluc(-/+) shCtrl/shSAPS3, **p*=0.038. f, siControl Gluc(+)/Gluc(-), **p*=0.036; siControl Gluc(-)/Gluc(-/+), ***p*=0.01; siSAPS1 Gluc(+)/Gluc(-), **p*=0.04; siSAPS1 Gluc(-)/Gluc(-/+), **p*=0.015; siSAPS2 Gluc(+)/Gluc(-), **p*=0.033; siSAPS2 Gluc(-)/Gluc(-/+) **p*=0.027; siSAPS3 Gluc(+)/Gluc(-), **p*=0.038; n.s., not significant.

Supplementary Fig.2 SAPS3 inhibits AMPK activity under metabolic stress

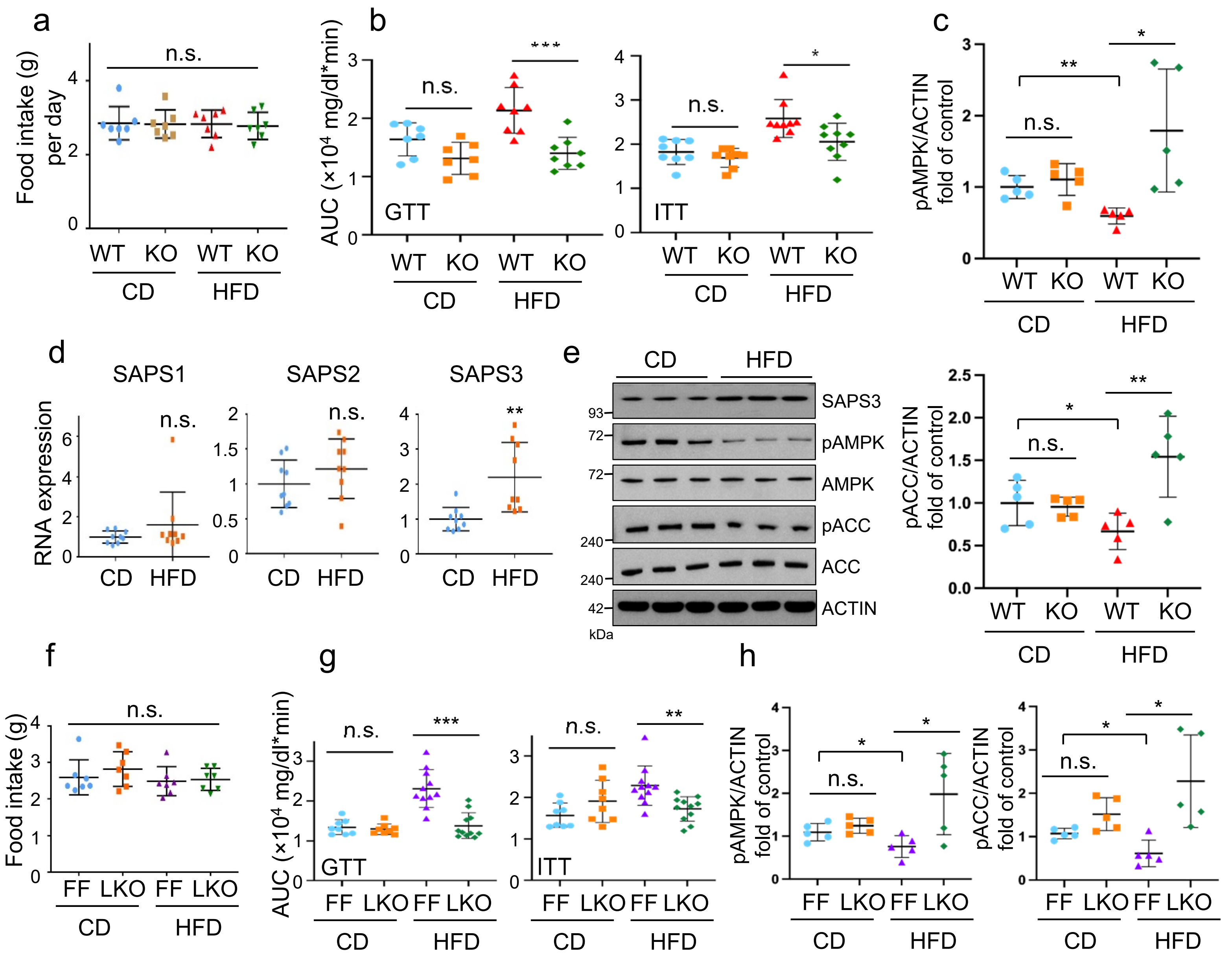

Supplementary Fig.2 SAPS3 inhibits AMPK activity under metabolic stress.
a Food intake was measured among different groups. Mean ± s.d., n=7 mice per group analyzed by one-way ANOVA. n.s., not significant. **b** Area under the curve (AUC) values of Fig.2e. Mean ± s.d., CD, n=7 mice per group; HFD, n=8 mice per group analyzed by two-tailed *t*-test, ***p=0.001, *p=0.035; n.s., not significant. **c** Quantification of western blot Fig.2j. Mean ± s.d., n=5 mice per group from two independent experiments analyzed by two-tailed *t*-test. pAMPK, **p*=0.035, ***p*=0.002; pACC, **p*=0.047, ***p*=0.01; n.s., not significant. **d** RNA expression of SAPS1, SAPS2 and SAPS3 in WT mice under HFD. Mean ± s.d., n=9 mice per group analyzed by two-tailed *t*-test. ***p*=0.006; n.s., not significant. **e** SAPS3 protein expression and AMPK activity in livers were analyzed by immunoblotting. The results are representative of two independent experiments. **f** Food intake per day was measured among different groups. Mean ± s.d., n=7 mice per group analyzed by one-way ANOVA. n.s., not significant. **g** Area under the curve (AUC) values of Fig.3h. Mean ± s.d., CD, n=7 mice per group; HFD, n=10 mice per group analyzed by unpaired *t*-test, ***p*=0.01, ****p*=0.0001. **h** Quantification of western blot Fig.3j was performed. Mean ± s.d., n=5 mice per group from two independent experiments analyzed by unpaired *t*-test. pAMPK, FF CD/HFD, **p*=0.041; pAMPK, HFD FF/LKO, **p*=0.041; pACC, FF CD/HFD, **p*=0.025; pACC, HFD FF/LKO, **p*=0.022; n.s., not significant.

Supplementary Fig.3 Loss of SAPS3 promotes fatty acid oxidation and inhibits fatty acid synthesis under HFD

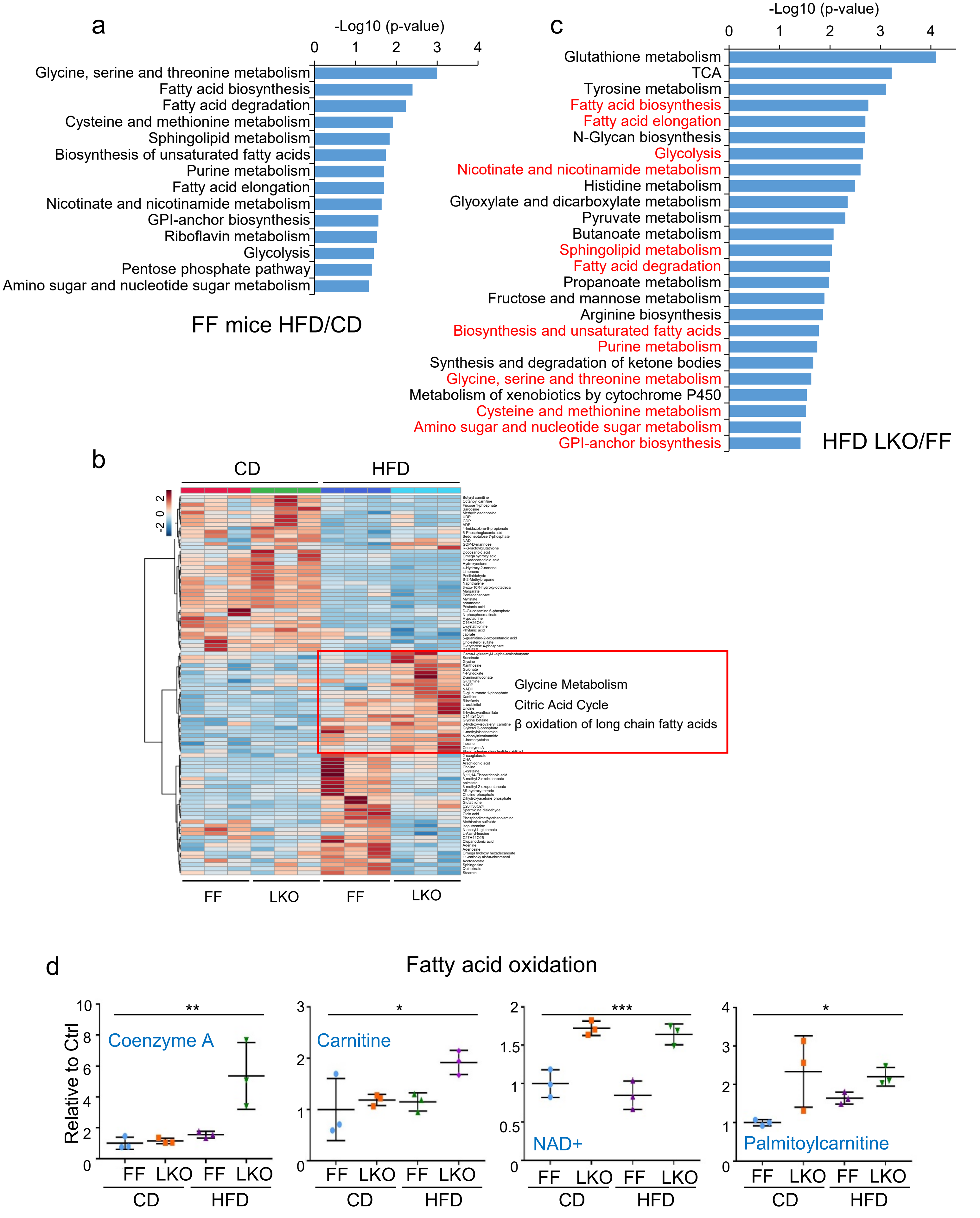

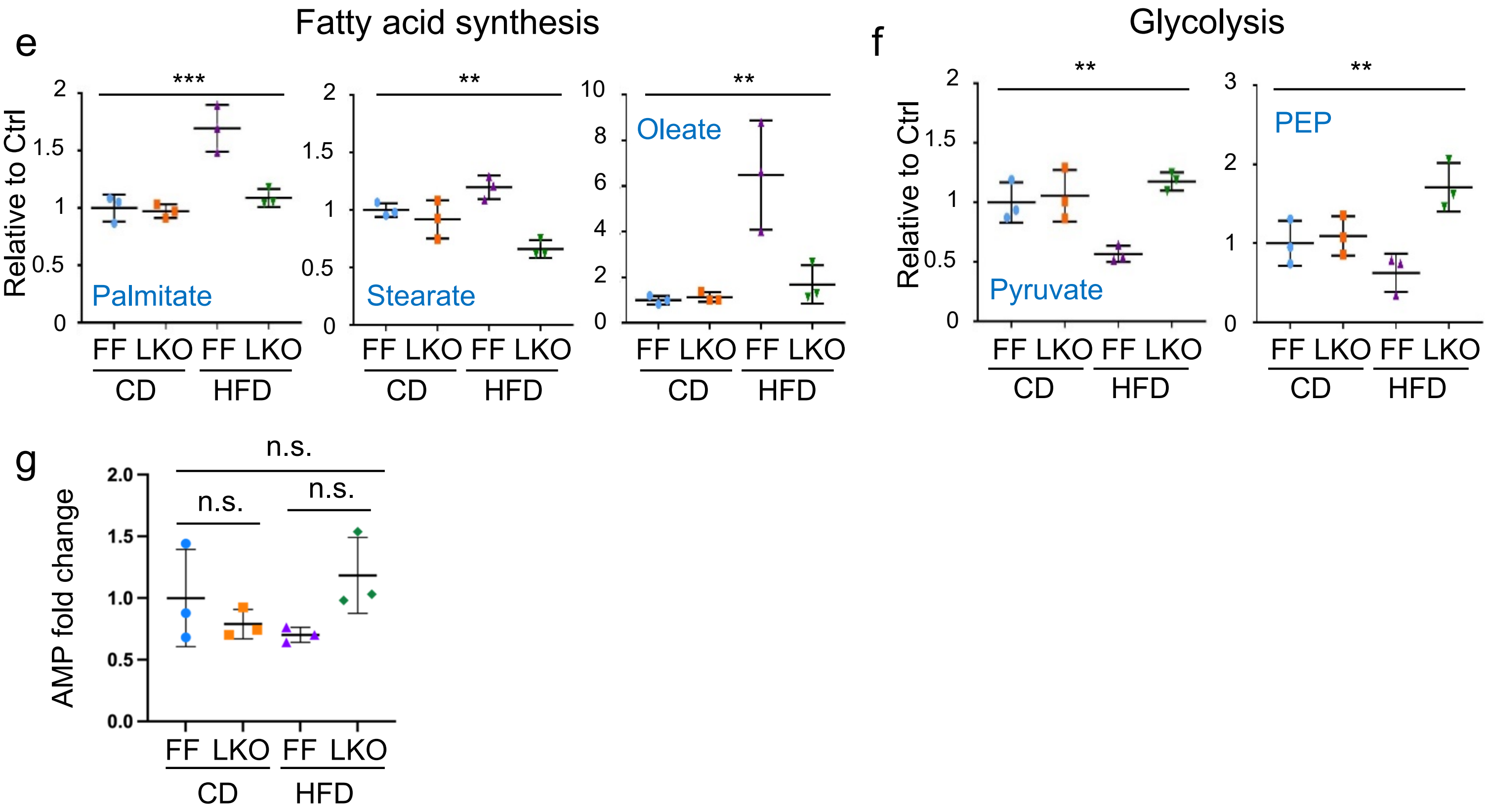

Supplementary Fig.3 Loss of SAPS3 promotes fatty acid oxidation and inhibits fatty acid synthesis under HFD.

a KEGG pathway analysis of metabolites from FF mice fed with CD and HFD. n=3 mice per group analyzed by unpaired *t*-test. **b** Pathway analyses of metabolites that did not change by HFD in WT mice but were significantly altered in HFD fed KO mice. **c** KEGG pathway analysis of metabolites in liver samples from FF and LKO mice fed with HFD. The pathways that were altered by HFD in FF mice were highlighted in red. n=3 mice per group analyzed by unpaired *t*-test. **d** The levels of coenzyme A, carnitine, NAD^+ and palmitoylcarnitine were compared to represent fatty acid oxidation rate. Mean ± s.d., n=3 mice per group analyzed by one-way ANOVA. Carnitine, *p=0.042; Palmitoylcarnitine, *p=0.036; **p=0.003; ***p=0.0002. **e** The levels of palmitate, stearate and oleate were compared to represent fatty acid synthesis rates. Mean ± s.d., n=3 mice per group analyzed by one-way ANOVA. Stearate, **p=0.002; Oleate, **p=0.002; ***p=0.0003. **f** The levels of pyruvate and PEP were compared to represent glycolysis rates. Mean ± s.d., n=3 mice per group analyzed by one-way ANOVA. Pyruvate, **p=0.004; PEP, **p=0.008. **g** The levels of AMP were compared to in different groups. PEP, phosphoenolpyruvate. Mean ± s.d., n=3 mice per group analyzed by one-way ANOVA or two-tailed *t* test, n.s., not significant.

Supplementary Fig.4 SAPS3 deletion reverses HFD altered genes expression

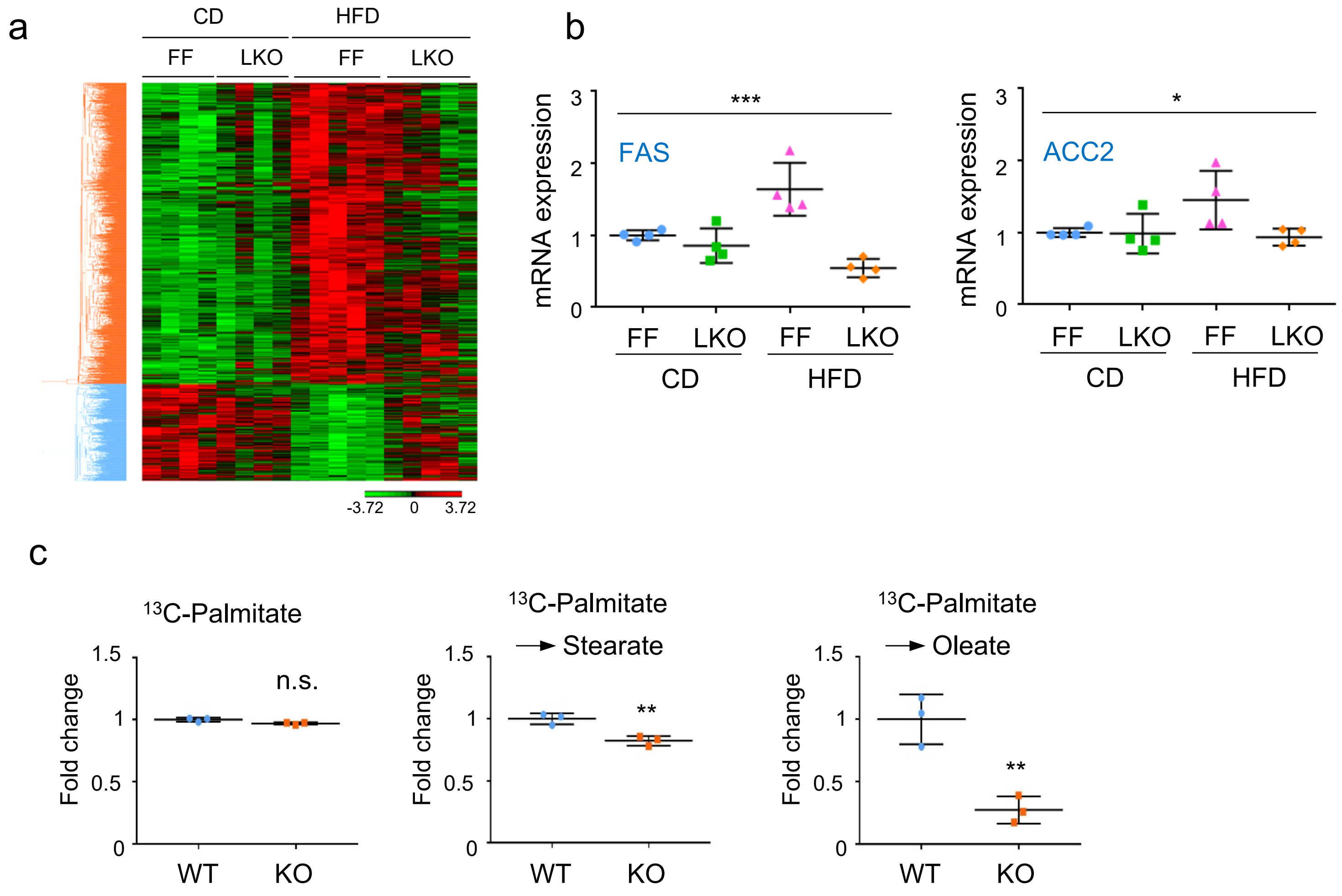

Supplementary Fig.4 SAPS3 deletion reverses HFD altered gene expression.
a Cluster of the 1780 genes that were significantly changed by HFD identified in (Figure 4C). **b** The expression levels of major fatty acid synthesis enzymes in liver samples. Mean ± s.d., n=4 mice per group analyzed by one-way ANOVA, **p*=0.011; ****p*=0.0001. FAS, fatty acid synthase; ACC2, acetyl-CoA carboxylase 2. **c** U-$^{13}C_{16}$ Palmitate and derived stearate and oleate levels in WT and SAPS3 KO MEF cells. Mean ± s.d., n=3 mice per group analyzed by two-tailed *t* test. Stearate, ***p*=0.006; Oleate, ***p*=0.01; n.s., not significant.

Supplementary Fig.5 SAPS3 regulates cellular metabolism via AMPK

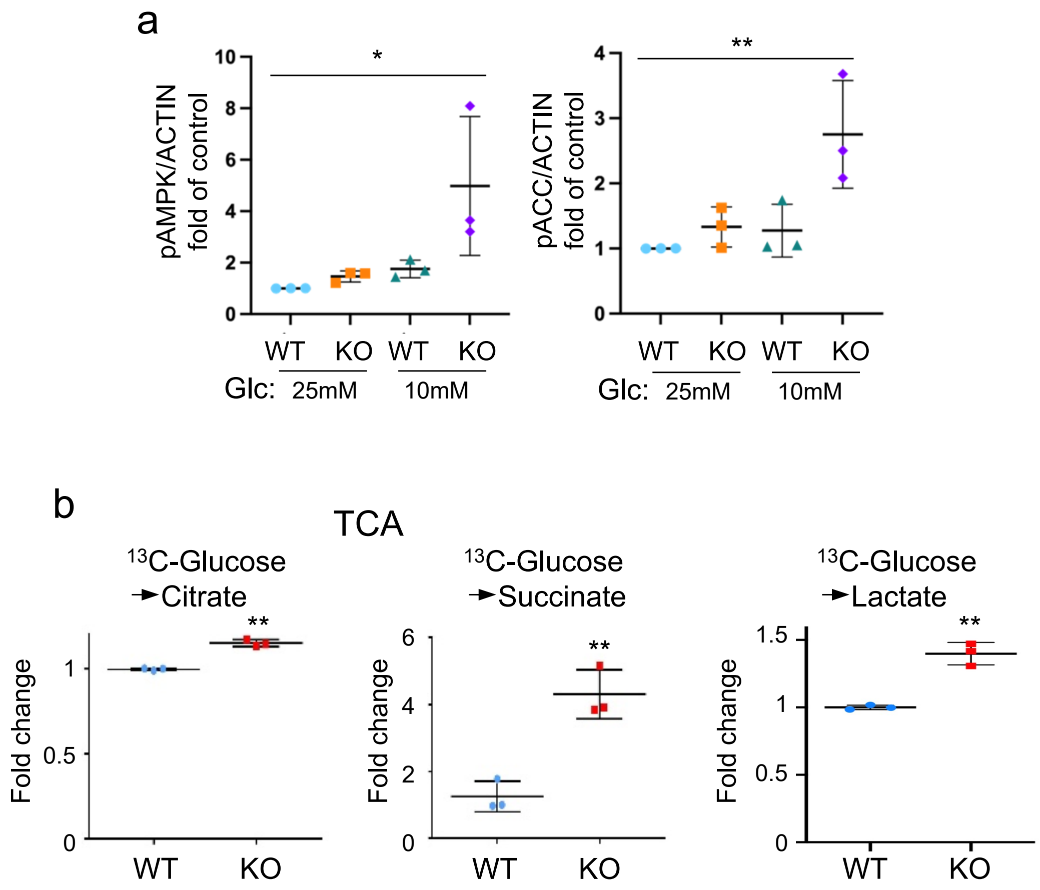

Supplementary Fig.5 SAPS3 regulates cellular metabolism via AMPK.
a Quantification of western blot Fig.5a. Mean ± s.d., n=3 biological replicates from three independent experiments analyzed by one-way ANOVA, *p=0.026; **p=0.008. **b** U-$^{13}C_6$ glucose-derived citrate, succinate, and lactate levels in WT and SAPS3 KO MEF cells. Mean ± s.d., n=3 biological replicates analyzed by two-tailed t test. Citrate, **p=0.002; Succinate, **p=0.006; Lactate, **p=0.0013.

Supplementary Fig.6 SAPS3 deletion promoted glucose homeostasis *in vivo* under HFD is AMPK dependent

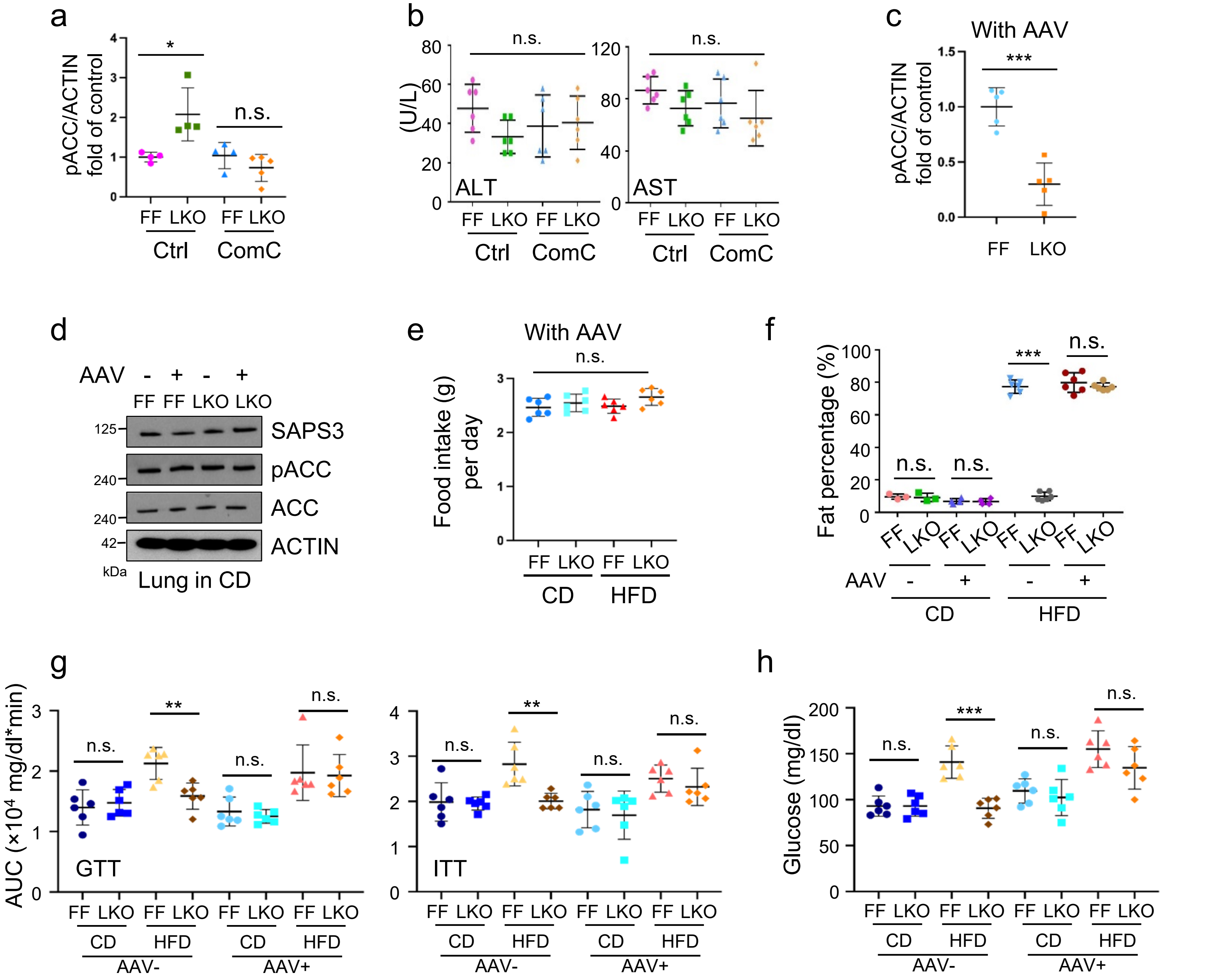

Supplementary Fig.6 SAPS3 deletion promoted glucose homeostasis *in vivo* under HFD is AMPK dependent.

a Quantification of western blot Fig.6d was performed. Mean ± s.d., n=4 or 5 mice per group from two different experiments analyzed by two-tailed *t*-test, **p*=0.045; n.s., not significant. **b** Liver functions were evaluated by the levels of ALT and AST. Mean ± s.d., n=6 mice per group analyzed by one-way ANOVA, n.s., not significant. **c** Quantification of western blot Fig.6h was performed. Mean ± s.d., n=5 mice per group from two independent experiments analyzed by two-tailed *t*-test, ****p*=0.0003. **d** Two weeks after the injection of AAV-AMPK-DN, lung samples were collected followed by immunoblotting with indicated antibodies. The results are representative of three independent experiments. **e** Food intakes were measured among different groups. Mean ± s.d., n=6 mice per group analyzed by one-way ANOVA, n.s., not significant. **f** The quantification of H&E staining images for Fig.6l. Mean ± s.d., CD, AAV (-) n=3 mice per group; CD, AAV (+) n=4 mice per group; HFD, AAV (-) n=6 mice per group; HFD, AAV (+) n=6 mice per group analyzed by two-tailed *t* test, ***p=1.7968E-10; n.s., not significant. **g** Area under the curve (AUC) values of Fig.6m. Mean ± s.d., n=6 mice per group analyzed by two-tailed *t*-test. GTT, **p=0.003; ITT, **p=0.007; n.s., not significant. **h** Fasting blood glucose level was measured. Mean ± s.d., n=6 mice per group analyzed by two-tailed *t*-test, ***p=0.0002; n.s., not significant.

Supplementary Figure 7. The uncropped images of all blots shown in the figures of this study.

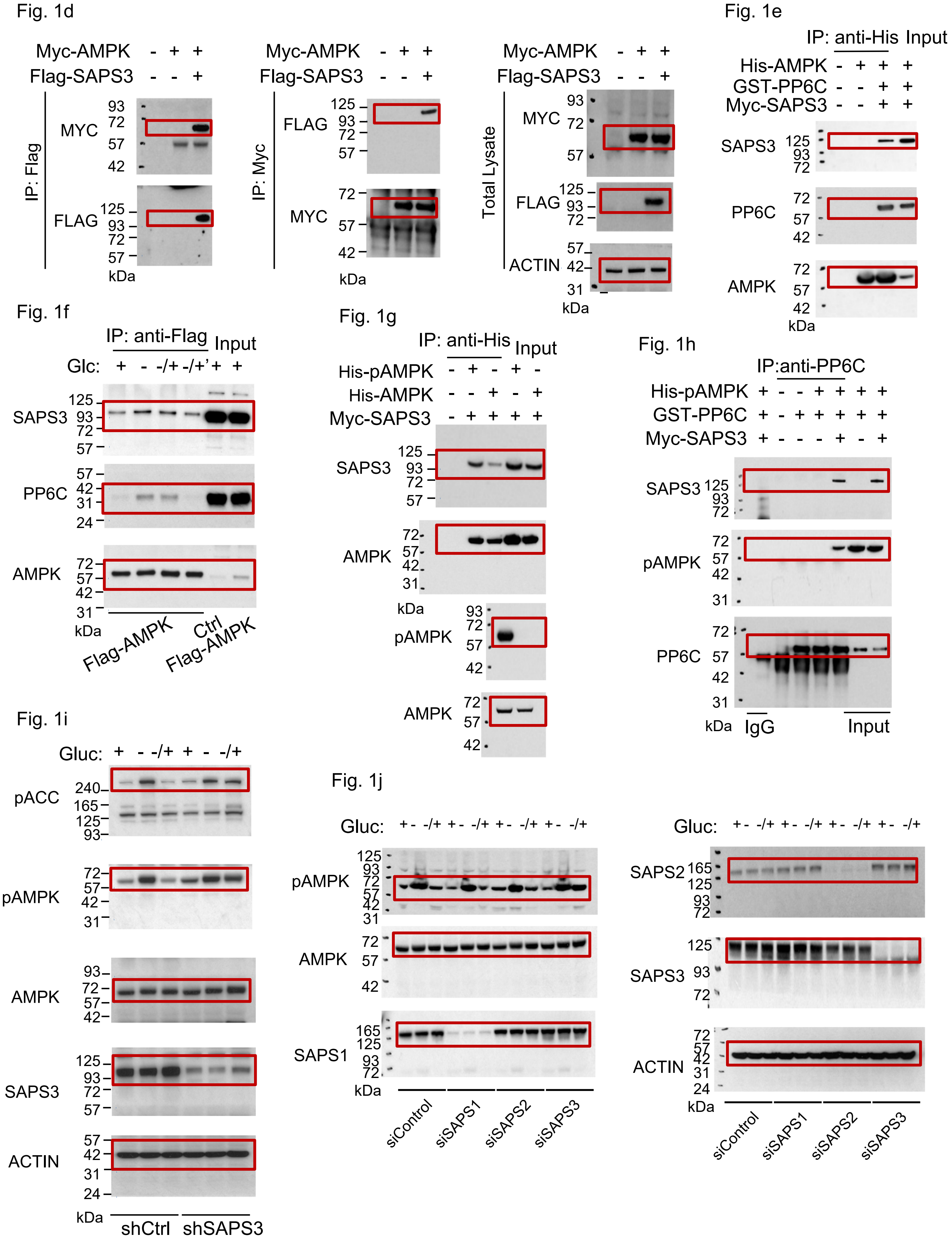

Supplementary Figure 7. The uncropped images of all blots shown in the figures of this study.

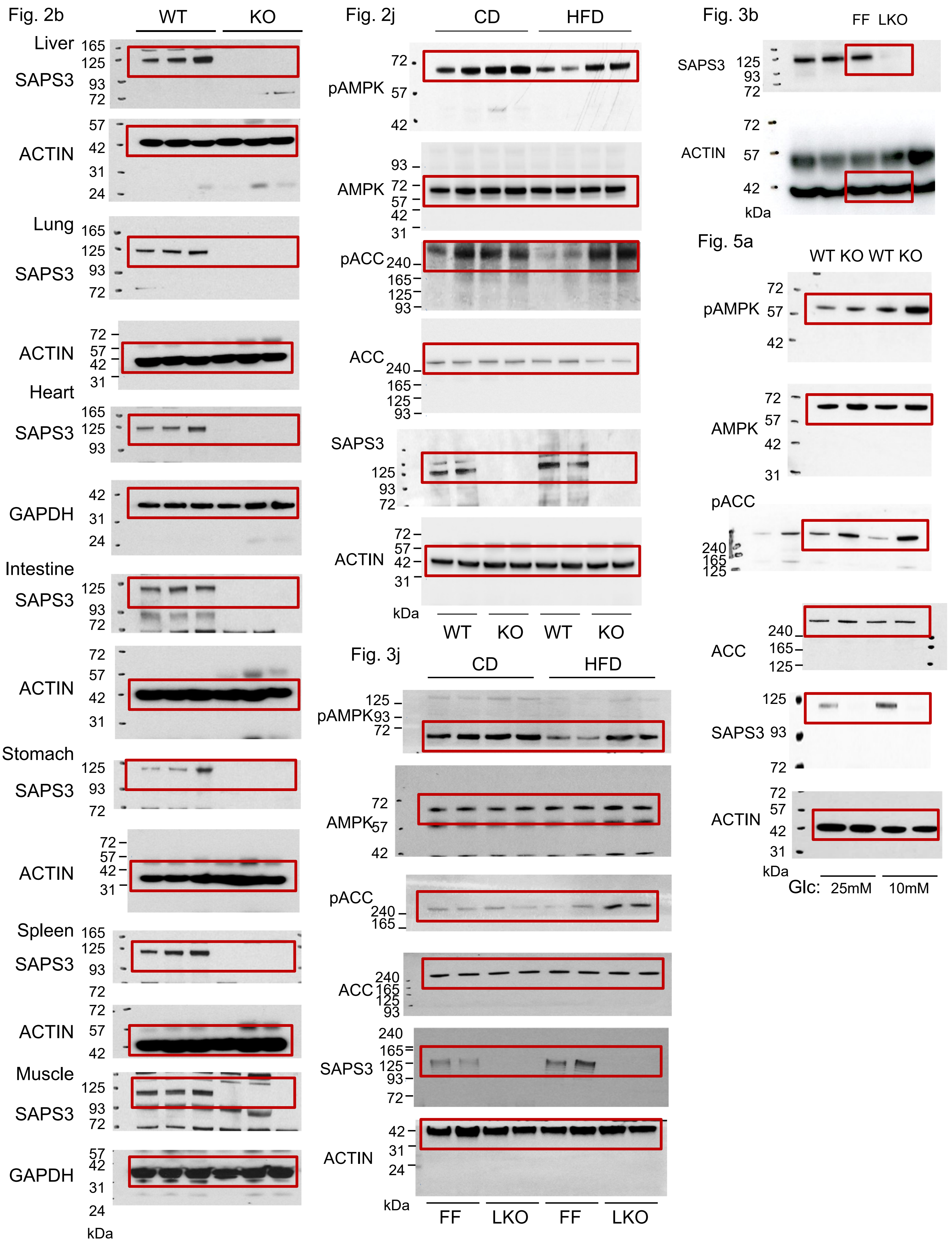

Supplementary Figure 7. The uncropped images of all blots shown in the figures of this study.

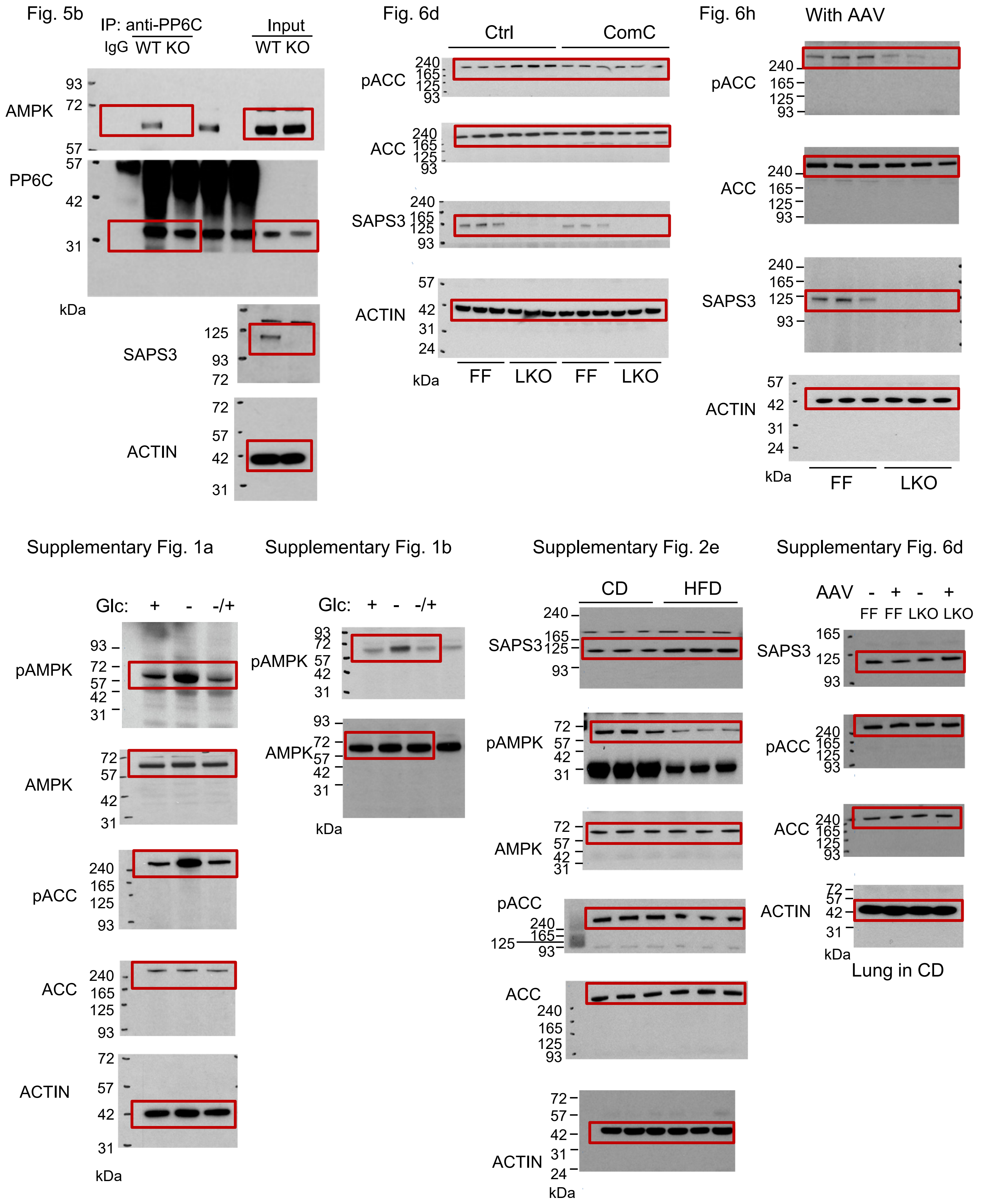

Supplement: Supplementary file 1 — Supplementary Information [file 41467_2023_36809_MOESM1_ESM.pdf]
