# Supplementary material for: SAPS3 subunit of protein phosphatase 6 is an AMPK inhibitor and controls metabolic homeostasis upon dietary challenge in male mice
Source: Nat Commun. 2023 Mar 13;14:1368. doi: 10.1038/s41467-023-36809-1 (PMC10011557; doi:10.1038/s41467-023-36809-1)
Supplement: Supplementary file 2 — Description of Additional Supplementary Files [file 41467_2023_36809_MOESM2_ESM.pdf]

Description of Additional Supplementary Files

File Name: Supplementary Data 1
Description: Raw data for metabolomics study before normalization
