# Supplementary material for: SAPS3 subunit of protein phosphatase 6 is an AMPK inhibitor and controls metabolic homeostasis upon dietary challenge in male mice
Source: Nat Commun. 2023 Mar 13;14:1368. doi: 10.1038/s41467-023-36809-1 (PMC10011557; doi:10.1038/s41467-023-36809-1)
Supplement: Supplementary file 4 — Reporting Summary [file 41467_2023_36809_MOESM4_ESM.pdf]

nature portfolio

Corresponding author(s): Mei Kong

Last updated by author(s): Jan 26, 2023

Reporting Summary

Nature Portfolio wishes to improve the reproducibility of the work that we publish. This form provides structure for consistency and transparency in reporting. For further information on Nature Portfolio policies, see our Editorial Policies and the Editorial Policy Checklist.

Statistics

For all statistical analyses, confirm that the following items are present in the figure legend, table legend, main text, or Methods section.

n/a	Confirmed	
☐	☒	The exact sample size (n) for each experimental group/condition, given as a discrete number and unit of measurement
☐	☒	A statement on whether measurements were taken from distinct samples or whether the same sample was measured repeatedly
☐	☒	The statistical test(s) used AND whether they are one- or two-sided<br>*Only common tests should be described solely by name; describe more complex techniques in the Methods section.*
☒	☐	A description of all covariates tested
☐	☒	A description of any assumptions or corrections, such as tests of normality and adjustment for multiple comparisons
☐	☒	A full description of the statistical parameters including central tendency (e.g. means) or other basic estimates (e.g. regression coefficient) AND variation (e.g. standard deviation) or associated estimates of uncertainty (e.g. confidence intervals)
☐	☒	For null hypothesis testing, the test statistic (e.g. F, t, r) with confidence intervals, effect sizes, degrees of freedom and P value noted<br>*Give P values as exact values whenever suitable.*
☒	☐	For Bayesian analysis, information on the choice of priors and Markov chain Monte Carlo settings
☒	☐	For hierarchical and complex designs, identification of the appropriate level for tests and full reporting of outcomes
☒	☐	Estimates of effect sizes (e.g. Cohen's d, Pearson's r), indicating how they were calculated

Our web collection on statistics for biologists contains articles on many of the points above.

Software and code

Policy information about availability of computer code

Data collection	qPCR-Bio-Rad CFX Maestro 1.0 was used to conduct the quantitative PCR.<br>LC-MS-ThermoScientific was used to collect LC-MS data.<br>Zeiss LSM 900 Airyscan confocal system was used to take IF images.<br>Agilent MassHunter was used for GC-MS data acquisition.
Data analysis	Microsoft Office Excel 365 ProPlus, GraphPad Prism 9, Seahorse Wave Desktop Software 2.4, LCMS Sieve 2.0, Image J 2.0.0, Agilent MassHunter qualitative and quantitative analysis, RNA-sequencing HISAT2 v2.0.4, Partek Genomic Suite v6.3 and Gene set enrichment analysis (GSEA) 4.0.

For manuscripts utilizing custom algorithms or software that are central to the research but not yet described in published literature, software must be made available to editors and reviewers. We strongly encourage code deposition in a community repository (e.g. GitHub). See the Nature Portfolio guidelines for submitting code & software for further information.

Data

Policy information about availability of data

All manuscripts must include a data availability statement. This statement should provide the following information, where applicable:

- Accession codes, unique identifiers, or web links for publicly available datasets
- A description of any restrictions on data availability
- For clinical datasets or third party data, please ensure that the statement adheres to our policy

The RNA-seq data generated in this study have been deposited in the Gene Expression Omnibus (GEO) under the accession code: GSE210333. The mass spectrometry proteomics data generated in this study have been deposited to the ProteomeXchange Consortium via the Proteomics IDEntifications Database (PRIDE) partner repository under the accession code: PXD039625. The metabolomics data generated in this study are provided in the Supplementary Table 1. Source Data are provided with this paper, and the uncropped gel/blot images are provided in the Supplementary Figure 7. All other data sets are available within the article and supplementary information.

Human research participants

Policy information about studies involving human research participants and Sex and Gender in Research.

Reporting on sex and gender	N/A
Population characteristics	N/A
Recruitment	N/A
Ethics oversight	N/A

Note that full information on the approval of the study protocol must also be provided in the manuscript.

Field-specific reporting

Please select the one below that is the best fit for your research. If you are not sure, read the appropriate sections before making your selection.

☒ Life sciences ☐ Behavioural & social sciences ☐ Ecological, evolutionary & environmental sciences

For a reference copy of the document with all sections, see nature.com/documents/nr-reporting-summary-flat.pdf

Life sciences study design

All studies must disclose on these points even when the disclosure is negative.

Sample size	For in vitro study, we did not perform statistical methods to predetermine sample size. The sample sizes were determined based on preliminary data or previous reports. All the experiments were repeated at least three independent times with technical triplicates that are commonly used sample sizes in the field. These sample sizes are sufficient to evaluate the effect of the treatment with low concentration of glucose. For in vivo study, the sample size were estimated based on preliminary data or for statistical power.
Data exclusions	No data were excluded from the analyses.
Replication	Biological and independent replicate experiments were successful. All in vitro experiments were repeated at least three independent times with triplicates. For in vivo experiments, 6 or more mice were used for each group.
Randomization	For in vivo experiments, animal fed with different diets were assigned randomly. Animals under different diets treated with control or compound C or AAV were divided randomly. For in vitro study, cells for different groups were treated randomly into experimental groups.
Blinding	For animal studies, all of the animal allocation and treatment were performed in a blinded manner. All the data collection and analyses were conducted blindly.

Reporting for specific materials, systems and methods

We require information from authors about some types of materials, experimental systems and methods used in many studies. Here, indicate whether each material, system or method listed is relevant to your study. If you are not sure if a list item applies to your research, read the appropriate section before selecting a response.

Materials & experimental systems

n/a	Involved in the study
☐	☒ Antibodies
☐	☒ Eukaryotic cell lines
☒	☐ Palaeontology and archaeology
☐	☒ Animals and other organisms
☒	☐ Clinical data
☒	☐ Dual use research of concern

Methods

n/a	Involved in the study
☒	☐ ChIP-seq
☒	☐ Flow cytometry
☒	☐ MRI-based neuroimaging

Antibodies

Antibodies used

anti-AMPKa, Clone# 34.2 (ab80039, Abcam), Dilution for WB 1:1000.
anti-pAMPKa, Clone# EPR5683 (ab133448, Abcam), Dilution for WB 1:1000.
anti-pAMPKa, Clone# D79.5E (4188, Cell signaling), Dilution for WB 1:1000.
anti-ACC (3662, Cell signaling), Dilution for WB 1:1000.
anti-pACC (Ser79) (3661, Cell signaling), Dilution for WB 1:1000.
anti-β-ACTIN, Clone# AC-15 (A1978, Sigma), Dilution for WB 1:5000.
anti-GAPDH, Clone# 14C10 (2218, Cell signaling), Dilution for WB 1:1000.
anti-SAPS3 (A300-971A, Bethyl), Dilution for WB 1:1000.
anti-PP6C (A300-844A, Bethyl), Dilution for WB 1:1000.
anti-MYC (ab9106, abcam), Dilution for WB 1:1000.
anti-FLAG Clone# M2 (F3165, Sigma), Dilution for WB 1:1000.
anti-SAPS3 (A300-972A, Bethyl), Dilution for WB 1:1000.
anti-SAPS1 (A300-968A, Bethyl), Dilution for WB 1:1000.
anti-SAPS2 (A300-969A, Bethyl), Dilution for WB 1:1000.
anti-AMPK Clone# 206CT5.4.2 (MA537501, Thermo Scientific), Dilution for IF 1:50.
and anti-SAPS3 (16944-1-AP, Thermo Scientific), Dilution for IF 1:100.
goat anti-rabbit 594 (Invitrogen, A11037), Dilution for IF 1:500.
goat anti-mouse 488 (Invitrogen, A11029, Dilution for IF 1:500.

Validation

Mouse antibodies:
anti-AMPKa [34.2] (ab80039, Abcam) reacts with rat, human, drosophila melanogaster, and mouse. The antibody is validated in human brain tissue lysate, HEK293 whole cell lysate, and mouse 3T3-L1 adipocytes whole cell lysate by Western blot based on manufacture's website.
anti-β-ACTIN [AC-15] (A1978, Sigma) reacts with human, mouse, rabbit, chicken and bovine. The antibody is validated in HeLa, 3T3 whole cell lysate, human foreskin fibroblasts, and rat liver protein lysate by Western blot based on manufacture's website.
anti-FLAG [M2] (F3165, Sigma) detects Flag fusion proteins. The antibody is validated in many publications. For example, PMID: 31266880, PMID: 24608790 and PMID: 22719065 by Western blot.
anti-AMPK [206CT5.4.2] (MA537501, Thermo Scientific) reacts with human. The antibody is validated in HeLa cells by Immunofluorecence, 293 whole cell lysate by Western blot based on manufacture's website.
Rabbit antibodies:
anti-pAMPKa [EPR5683] (ab133448, Abcam) reacts with mouse, rat, human and drosophila melanogaster. The antibody is validated in mouse heart lysate and HEK293 whole cell lysate by Western blot based on manufacture's website.
anti-pAMPKa [D79.5E] (4188, Cell signaling) reacts with human, mouse and rat. The antibody is validated in many publications. For example, PMID: 36167800, PMID: 36230981 and PMID:36046628 by Western blot.
anti-ACC (3662, Cell signaling) reacts with human, mouse and rat. The antibody is validated in 293, HeLa and 3T3 cell lysate by Western blot based on manufacture's website.
anti-pACC (Ser79) (3661, Cell signaling) reacts with human, mouse and rat. The antibody is validated in HEK293 whole cell lysate by Western blot based on manufacture's website.
anti-GAPDH [14C10] (2218, Cell signaling) reacts with human, mouse and rat. The antibody is validated in HeLa, 3T3, C6 and L929 cell lysate by Western blot based on manufacture's website.
anti-SAPS3 (A300-971A, Bethyl) reacts with human and mouse. The antibody is validated in HeLa cells by Western blot/ Immunoprecipitation based on manufacture's website.
anti-PP6C (A300-844A, Bethyl) reacts with human and mouse. The antibody is validated in HeLa and mouse 3T3 whole cell lysate by Western blot/ Immunoprecipitation based on manufacture's website.
anti-MYC (ab9106, abcam) detects Myc fusion proteins. The antibody is validated in Myc tagged cdc25 overexpressed 293FT whole cell lysate by immunoprecipitatioin based on manufacture's website.
anti-SAPS3 (A300-972A, Bethyl) reacts with human. The antibody is validated in HeLa whole cell lysate by Western blot based on manufacture's website.
anti-SAPS1 (A300-968A, Bethyl) reacts with human. The antibody is validated in HeLa whole cell lysate by Western blot based on manufacture's website.
anti-SAPS2 (A300-969A, Bethyl) reacts with human. The antibody is validated in HeLa and 293T whole cell lysate by Western blot based on manufacture's website.
anti-SAPS3 (16944-1-AP, Thermo Scientific) reacts with human, mouse and rat. The antibody is validated in HepG2 by immunoprecipitatioin based on manufacture's website.

Eukaryotic cell lines

Policy information about cell lines and Sex and Gender in Research

Cell line source(s)	293T cells (ATCC® CRL-3216™) were derived from human fetus and HT1080 cells (ATCC® CCL-121™) were derived from a male human patient.
Authentication	Cells purchased from ATCC were authenticated by ATCC. The cells were authenticated by STR profiling analysis.
Mycoplasma contamination	Cells were tested for mycoplasma using MycoAlert mycoplasma detection kit. All cells in this manuscript were tested negative.
Commonly misidentified lines (See ICLAC register)	No, we did not use commonly misidentified lines.

Animals and other research organisms

Policy information about studies involving animals; ARRIVE guidelines recommended for reporting animal research, and Sex and Gender in Research

Laboratory animals	Eight weeks old male C57BL/6J mice (000664, Jackson Lab) and male ppp6r3fl/fl C57BL/6J mice were used in this manuscript. All animals were maintained under specific pathogen-free conditions. These mice were housed in a 12-h-light/12-h-dark cycle at a temperature controlled (23°C±0.9°C) with 50%±4% humidity, facility with free access to food and water.
Wild animals	The study did not involve wild animals.
Reporting on sex	Male mice were used in this study based on the reports indicating that estrogens protect against high fat diet-induced detrimental effect in female rodents (PMID: 19164473).
Field-collected samples	No field collected samples in this manuscript.
Ethics oversight	All studies involving animals were performed according to approved IACUC protocol (AUP-20-159) at the University of California, Irvine.

Note that full information on the approval of the study protocol must also be provided in the manuscript.
